# TriPepSVM: *de novo* prediction of RNA-binding proteins based on short amino acid motifs

**DOI:** 10.1093/nar/gkz203

**Published:** 2019-03-29

**Authors:** Annkatrin Bressin, Roman Schulte-Sasse, Davide Figini, Erika C Urdaneta, Benedikt M Beckmann, Annalisa Marsico

**Affiliations:** 1 Max Planck Institute for Molecular Genetics, Ihnestrasse 63-73, 14195 Berlin, Germany; 2 IRI Life Sciences, Humboldt University Berlin, Philippstrasse 13, 10115 Berlin, Germany; 3 Free University of Berlin, Takustrasse 9, 14195 Berlin, Germany; 4 Institute of Computational Biology (ICB), Helmholtz Zentrum Munich, Ingolstaedter Landstr. 1 85764 Neuherberg, Germany

## Abstract

In recent years, hundreds of novel RNA-binding proteins (RBPs) have been identified, leading to the discovery of novel RNA-binding domains. Furthermore, unstructured or disordered low-complexity regions of RBPs have been identified to play an important role in interactions with nucleic acids. However, these advances in understanding RBPs are limited mainly to eukaryotic species and we only have limited tools to faithfully predict RNA-binders in bacteria. Here, we describe a support vector machine-based method, called TriPepSVM, for the prediction of RNA-binding proteins. TriPepSVM applies string kernels to directly handle protein sequences using tri-peptide frequencies. Testing the method in human and bacteria, we find that several RBP-enriched tri-peptides occur more often in structurally disordered regions of RBPs. TriPepSVM outperforms existing applications, which consider classical structural features of RNA-binding or homology, in the task of RBP prediction in both human and bacteria. Finally, we predict 66 novel RBPs in *Salmonella* Typhimurium and validate the bacterial proteins ClpX, DnaJ and UbiG to associate with RNA *in vivo*.

## INTRODUCTION

Gene regulation in eukaryotes occurs at several levels and involves the action of transcription factors, chromatin, RNA-binding proteins (RBPs) and other RNAs. RBPs and messenger RNAs (mRNAs) form ribonucleoprotein complexes (RNPs) by dynamic, transient interactions, which control different steps in RNA metabolism, such as RNA stability, degradation, splicing and polyadenylation. Furthermore, numerous diseases like neuropathies, cancer and metabolic disorders have also been linked to defects in RBPs expression and function (1–3).

Interactome Capture (RIC) has enabled proteome-wide identifications of RBPs (4). RIC utilizes UV cross-linking to induce stable RNA-protein interactions in living cells, followed by poly(A) RNA selection via magnetic oligo d(T) beads and subsequent protein identification by mass-spectrometry. RIC studies yielded hundreds of novel RBPs in e.g. human HeLa (5), HEK293 (6), Huh-7 (7) and in K562 (8) cells but also in worm and yeast (7,9), which do not harbor canonical RNA-binding domains (RBDs), as well as factors which were not previously associated with RNA biology. Among them we find enzymes, cell cycle regulators and dual specificity DNA–RNA binders, including transcription factor and chromatin components (3). The discovery of these unconventional RBPs without known RNA-binding motifs suggests the existence of new modes of RNA binding and the involvement of RBPs in previously unexplored biological processes (10).

Intrinsically disordered regions (IDRs) are widespread in the proteome and have been shown to be involved in regulatory functions, including direct RNA binding (11). RBPs identified by RIC are highly enriched in disordered regions compared to the whole human proteome and are characterized by low complexity, repetitive amino acid sequences. In particular, a low content of bulky hydrophobic amino acids and a prevalence of small, polar and charged amino acids are found in unstructured regions of RBPs. These amino acids, such as glycine (G), arginine (R) and lysine (K), as well as the aromatic residue tyrosine (Y), form shared sequence patterns among RBPs. For example RGG box binding motifs and glycine/tyrosine boxes YGG are broadly used platforms for RNA binding and can work alone or in combination with classical RBDs (11).

The occurrence of IDRs within RBPs appears to be conserved from yeast to humans. In earlier work, we defined a core set of RBPs conserved from yeast to human and identified [K]- and [R]-rich tripeptide repeat motifs as conserved across evolution, making IDRs plastic components of RBP co-evolution (7). Importantly, we proposed that the number of repeats in IDRs of RBPs considerably expands from yeast to human, while the number of RBDs remains the same, linking repeat motifs in RBP IDRs to the functional complexity of regulation in higher eukaryotes.

Research over the past two decades has revealed extensive post-transcriptional control in bacteria as well, with regulatory networks comprising RBPs and small non-coding RNAs (sRNAs). Bacterial RBPs are relatively simple, often possessing only a few or even a single RBD per protein, which recognizes a short RNA sequence (12). This is unlike eukaryotic RBPs, whose modular architecture enables versatility and combinatorial RBP-RNA interactions. Classical RBDs, such as the S1 domain, cold-shock domain, Sm and Sm-like domains, the double-stranded RNA binding domain, the K-homology domain and others, are widespread among bacteria (12). One third of annotated bacterial RBPs are ribosomal proteins. Other bacterial RBPs are involved in regulatory functions of transcription termination, RNA synthesis, modification and translation, like the well-known Hfq protein. The latter is a protein platform to mediate mRNA:sRNA interaction in Gram negative bacteria and the protein was shown to have several modes of RNA interaction, involving translational inhibition and repression, protection and induction of degradation by RNases or priming mRNAs for polyadenylation and subsequent degradation (13,14).

Since interactome capture relies on the use of oligo(dT) to isolate proteins bound to mRNA, the method is not applicable to bacterial species which generally lack polyadenylation (except for degradation purposes). Although experimental approaches have been developed recently to extend RBP catalogs to include RBPs not identified by RIC techniques (15,16), bacterial RBPs remain poorly annotated. Therefore, computational approaches able to predict new RBPs in both pro- and eukaryotes in a proteome-wide manner are in high demand, in order to identify putative candidate RBPs for further experimental investigation. Several *in silico* methods have been developed to predict RBPs from primary sequence and/or protein structure (17–20). Approaches that characterize RBPs by predicting RNA binding residues from known protein-RNA structures are computationally expensive (21). Further, they can only be trained on the small subset of RBPs for which the structure is known, therefore performing well only on specific datasets (20,21). Such methods do not generalize well to proteins whose structure is still unknown, which is the case for most bacterial RBPs.

Computational prediction tools such as SPOT-Seq (17), RNApred (22), RBPPred (19), catRAPID (18) and APRICOT (20), either derive sequence-structure features such as biochemical properties and evolutionary conservation and train a supervised classifier to distinguish RBPs from non-RBPs or classify proteins based on whether they harbor a known RBD or other RNA binding motifs (1). However, *in silico* methods that identify RBPs based on known domains might have some limitations, as one third of RBPs from recent experimental studies do not have prior RNA-binding related homology or domain annotation (5,7). Therefore such methods might generate a high percentage of false negatives, i.e. RNA-binders which lack an RBD and therefore are not predicted as such, as well as false positive, RBPs with classified RBDs that perform non-RNA binding functions (3). A recently developed method, SONAR, exploits the fact that proteins that interact with many others RBPs from a protein-protein interaction (PPI) network are more likely to be RBPs themselves (23). Although SONAR is also suitable for the prediction of ‘unconventional’ RBPs, it heavily depends on the quality and depth of the underlying PPI network.

The many newly characterized RBPs, their nontypical RBDs (or the lack of them), as well as the observation that IDRs are subject to strong sequence constraints under the form of conserved amino acid triplets conserved in the RBPs, prompted us to explore the possibility that RBPs might be confidently predicted based purely on the occurrence of short amino acid *k*-mers.

We set up to predict whether a protein is likely to be an RBP or not based on primary sequence using a string kernel (spectrum kernel) support vector machine (SVM). The spectrum kernel in combination with SVMs was first successfully introduced by Leslie *et al.* in the context of protein family classification (24). Support vector machine classifiers have been used successfully in other biological tasks, for example the identification of specific regulatory sequences (25) and in RBP prediction as well (19).

In this paper, we describe our newly developed RBP prediction method TriPepSVM. It applies for the first time string kernel support vector machines for RBP prediction. The model uses exclusively k-mer counts to classify RBPs versus non-RBPs in potentially any species. It does not depend on any prior biological information such as RBD annotation or structure, and therefore allows RBP prediction in an unbiased manner. We show that TriPepSVM performs better than other methods in RBP prediction in human and the bacteria *Escherichia coli* and *Salmonella* Typhimurium. In addition, it also reliably predicts RBPs across related species. Our method recovers RBPs characterized previously in RIC studies with high sensitivity and finds both, RBPs adopting classical RNA-binding architectures as well as RBPs lacking known RBDs.

## MATERIALS AND METHODS

### Data sets

In the following, we describe the data collection pipeline to derive the data sets for training and evaluating TriPepSVM on human, *E. coli* and *Salmonella*. It is based on the UniprotKB database (26) and derives data automatically for a given taxon (see Supplementary Figure S1). We used the taxon identifier 9606 to collect data for *Homo sapiens*, taxon identifier 590 for the *Salmonella* clade and the identifier 561 for *E. coli*.

#### Collection of annotated RNA-binding proteins (positive set)

We utilize the gene ontology database QuickGO (27) to collect annotated RBPs from UniprotKB. We apply the term *RNA-binding* (GO:0003723), also including all associated sub-terms, i.e. *tRNA binding, snRNA binding* and *poly(A) RNA binding*. The number of annotated RBPs in the QuickGO database is limited for some organisms, therefore our pipeline also supports a *recursive mode* to collect positive data from all members of a specified taxon or branch. For example, taxon 590 will retrieve annotation for all *Salmonella* strains. In order to avoid duplicate annotations or paralogs in UniprotKB, the software CD-Hit (28) was used to remove proteins with a sequence similarity higher than 90%.

This way, we collect 1812 known RBPs for human, 306 for *Salmonella* and 512 for *E. coli*.

#### Collection of non-RNA-binding proteins (negative set)

Since it is challenging to define a non-RNA-binding protein, we developed a strict filtering to generate the negative set for our method. We first collect the whole Swiss-Prot proteome of a given taxon and then remove all nucleotide-binding proteins in a step-wise manner. First, we remove proteins with an amino acid sequence length smaller than 50 AA or greater than 6000 AA. Short sequences result in very sparse representations of *k-mer* count vectors for SVM training while very long sequences might bias the SVM learning. Secondly, we utilize the Uniprot keyword database and QuickGO annotations to remove annotated nucleotide-binding proteins (both filtering steps are adopted from (29); see full list in Supplementary Table S4 and Table S5). Finally, we discard proteins containing at least one annotated or potential RBD from collected Pfam (30) domains (see Supplementary Table S6). Similarly to the positive set, CD-Hit was used to remove redundant protein sequences.

We obtain 12 038 non-RNA-binding proteins for human, 1415 for *Salmonella* and 3783 non-RNA-binders for *E. coli*.

#### Independent validation set from RIC studies

We collected a set of experimentally confirmed RBPs from three independent interactome capture studies (6–8) and from a review on RBPs (3) to evaluate the sensitivity of our model in human. First, we excluded protein sequences that were already present in our training data set. Secondly, we evaluated TriPepSVM on the human proteome independently for all four data sets and then on their union. The sensitivity was computed as the fraction of experimentally detected RBPs which are also predicted by our model.

### TriPepSVM prediction model

**Figure 1.**
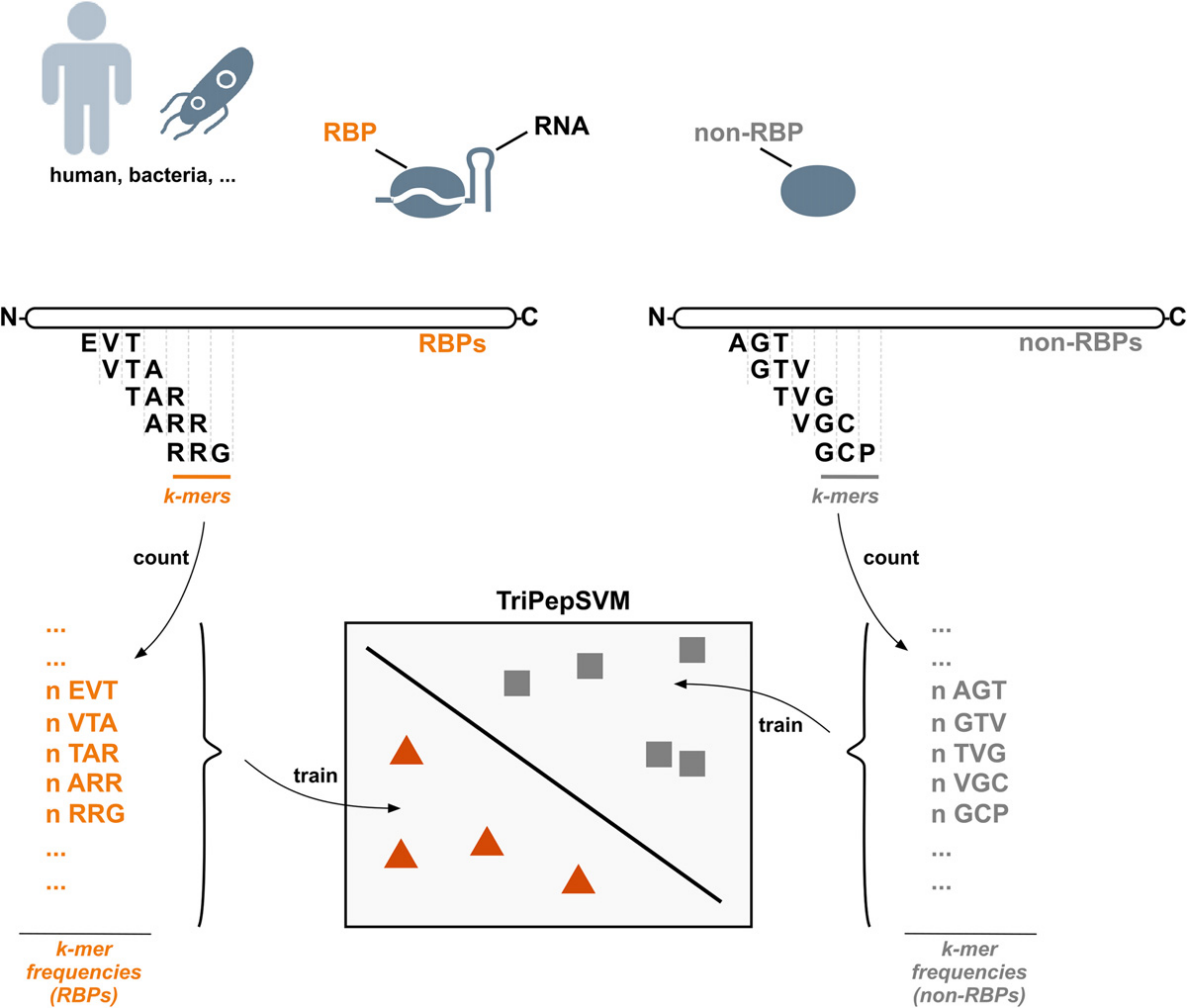
TriPepSVM schematic. TriPepSVM is a support vector machine trained on tri-peptide frequencies from RBPs and non-RBPs to discriminate between these two proteins. A protein-sequence is split into a set of overlapping *k*-mers. All of the k-mers of a sequence form a vector which is then fed into the SVM classifier that learns a decision boundary to separate RBPs from non-RBPs.

TriPepSVM is a discriminative machine learning model based on Support Vector Machines (SVMs) which is trained to classify RBPs versus non-RBPs based on sequence content alone. SVMs are a class of supervised learning algorithms which, given a set of labelled training vectors (positive and negative input examples) learn a linear decision boundary by finding the optimal hyperplane to discriminate between the two classes (31). The result is a linear classification rule that can be used to classify new test points, in our case new protein sequences, into one of the two classes, RBP or non-RBP (see Figure 1). When using a kernel in conjunction with an SVM, input points are implicitly mapped into a high-dimensional vector space where coordinates are given by feature values. The SVM produces then a linear decision boundary in this high-dimensional space. In our model we use the spectrum kernel (24) for classification, a linear kernel that allows the application of SVMs to strings (and therefore to amino acid sequences). Given a number *k* ≥ 1, the *k*-spectrum of an input sequence is the set of all the *k*-length (continuous) sequences, also called *k*-mers, that it contains (see figure 1). The high-dimensional feature representation for a sequence *x*, Φ(*x*), is then a vector where each entry counts the number of times each *k*-mer, from a pool of |Σ|^*k*^ possible *k*-mers from an alphabet of size Σ, occurs in the given sequence. The *k*-spectrum kernel is denoted as the dot product between two sequence feature vectors:

(1) \begin{equation*} K_k(x,y)=<\Phi _k(x), \Phi _k(y)>\end{equation*}

#### Model training

We randomly split the collected data into training (90%) and testing (10%) data sets, and stratified the data such that training and test data contain both roughly the same ratio of positives to negatives (see Table 1 for the exact sizes of training and test data sets for all organisms).

**Table 1.** Sizes of training and test sets for all three organisms

	Training		Test		
Data Set	Pos	Neg	Pos	Neg	Pos. Ratio
**Human**	1625	10834	181	1204	15%
***Salmonella***	275	1273	31	142	22%
***E. coli***	460	3404	52	379	14%

As both, training and test sets are heavily imbalanced, i.e. they contain many more negatives than positives, we chose a class-weighted SVM (32) approach, which accounts for class imbalance by weighting the cost parameter *C* differently for positive and negative data points. The class-weighted SVM was used together with the spectrum kernel from the *KeBABS*-package in *R* (33). To obtain the optimal values of the hyperparamters *k*, the sub-string length and *C*, the SVM cost parameter, we perform cross-validation, splitting the data into *n* equally sized subsets as illustrated in Supplementary Figure S2. We also run a separate ‘outer’ loop to select the optimal combination of weights *W* + and *W* − for the positive and the negative class, respectively (see Supplementary Paragraph 1.3.3 and 2.1 for more details).

For comparison, and to better asses the effect of the imbalanced dataset on TriPepSVM performance, we applied a bootstrap approach and alternatively train our model on balanced data by randomly sub-sampling the negative set 10 times to match the size of the positive set. We then average the performance on the test set over the 10 runs (as described in detail in Supplementary Paragraph 2.3).

#### Feature importance scores

Since the spectrum kernel ϕ is a linear kernel, we can obtain meaningful feature weights from the solution of the SVM optimization problem, similar to a linear regression scenario. The weight vector $\boldsymbol{w}$ for all *k*-mers, whose entries are the single *k*-mer contributions to the classification problem can be computed as:

(2) \begin{equation*} \boldsymbol{w} =\left( \sum _{i=1}^{n} y_i\alpha _i\phi _{k\mbox{-}mer} (\boldsymbol{x}_i) \right)_{k\mbox{-}mer \in |\Sigma |^{k}} \end{equation*}

for *n* points $(\boldsymbol{x_i},y_i)$, where $x_i \in \mathbb {R}^{m}$ is a *m*-dimensional data point and *y*_*i*_ ∈ {0, 1} is the associated label. The values of $\boldsymbol{\alpha }$ are the results from the SVM optimization problem, and contain non-zero values for important data points, the so-called *support vectors*, which are the points closest to the SVM decision boundary (24).

Important *k*-mers show large absolute values in $\boldsymbol{w}$ and the sign of the weight indicates to which class, positive or negative, a *k*-mer contributes to.

### Application of existing methods for RBP classification

We apply different RBP prediction tools for the performance comparisons. We focus on approaches that allow for proteome-wide predictions, such as SPOT-Seq-RNA, RNAPred and RBPPred. We excluded catRAPID because the web-server does not allow submission of >100 protein sequences, making proteome-wide predictions not feasible in this setting.

#### Pfam-domain-recognition

We collect profile Hidden Markov Models (HMMs) for known RBDs from the Pfam database (PFAM 27.0). We obtain 219 different HMMs annotated as ‘RNA binding/recognition’ in the Pfam description, quickGO or Protein Data Bank (34). We then use the HMMER software (35) for scanning the proteomes for proteins containing at least one of the collected RBDs. We run HMMER with default parameters, i.e. using an *E*-value cutoff ≤10.

#### SPOT-Seq-RNA

SPOT-Seq-RNA is a template-based technique to predict RNA-binding potential of a query protein (17). It employs a library of non-redundant protein-RNA complex structures and attempts to match a query sequence to the protein structure in the protein–RNA complexes by fold recognition. More specifically, SPOT-Seq-RNA requires a protein sequence in FASTA format, it passes it to PSI-BLAST to search for homologous sequences and to generate a position-specific scoring matrix (PSSM). The PSSM is used to predict several structural properties and the structural profile is matched against the known templates to compute a matching score (*Z*-score). Statistically significant matching templates with low free energy of the RBP-RNA complex are then used in order to assign a putative RNA-binding function to the query protein. We used a local version of the tool, with *E*-*value* < 0.001 from PSI-BLAST, a minimum template matching *Z*-score of at least 8.04 and maximum binding free energy of –0.565, as proposed by the authors.

#### RNApred

RNApred is an SVM-based approach to predict RBPs (22) and its application supports three different modes based on: (i) amino acid composition only, (ii) evolutionary information under the form of a PSSM built from PSI-BLAST and (iii) a combination of the previous two modes with some additional refinements. Unfortunately mode (i) is the only one that can be efficiently applied proteome-wide from the RNApred web server (as the other two modes only supported submission of one protein sequence per time) and therefore mode (i) was chosen.

#### RBPPred

RBPPred is also based on Support Vector Machines for the classification task of RBPs versus non-RBPs (19). Compared to the other earlier tools, it uses comprehensive feature representation which includes protein physiochemical properties, as well as evolutionary information under the form of a PSSM derived from PSI-BLAST, similarly to SPOT-Seq-RNA. We downloaded the command line version of RBPPred from the author’s website and applied it on our test data set.

### Performance metrics

The performance of all methods was evaluated using two metrics: the area under the receiving operating characteristics curve (AUROC) and the area under the precision-recall curve (AUPR). Both metrics require a set of proteins which have been scored according to their likelihood of being an RBP or not, as well as the known protein class. However, PR curves give a better idea of the false discovery rate of each method as they can better account for class imbalance (36) compared to ROC curves (see Supplementary Paragraphs 1.3.1 and 1.3.2 for more details). Most of the evaluated methods, except for SPOT-Seq-RNA, return a score or probability for each protein to be RNA-binding and therefore could be assessed via these two metrics.

We also computed sensitivity, specificity, precision, balanced accuracy and Matthews correlation coefficient (37) (MCC, see Supplementary Figure S4) for the different methods, using the optimal cutoff to distinguish RBPs from non-RBPs from the PR curve, as described in Supplementary Paragraph 1.4.

### Classification of tripeptides in ordered/disordered protein regions

We analysed the relative abundance of all tripeptides in structurally disordered versus ordered regions of RBPs with the IUPred tool (38), which allows to characterize disordered protein regions lacking well-defined tertiary structure. IUPred provides a mode to predict globular domains with an average disorder score smaller than 0.5. We used this mode to classify each amino acid as ‘structured’ if it occurred in a predicted domain and disordered if it did not. From there, we were able to estimate the fraction of structural disorder for each tripeptide defined as the number of times that the tripeptide was found in disordered regions of RBPs divided by the number of times the same tripeptide was found in RBPs.

### Cell culture and strains

*Salmonella enterica* subsp. *enterica* serovar Typhimurium strain SL1344 was cultivated in standard LB medium if not stated otherwise. We generated chromosomal insertions of FLAG-encoding sequences downstream of candidate genes using the Lambda Red technique (39,40). For details see Supplementary Methods Paragraph 1.5.

### Molecular biology techniques

#### Western blotting

We performed western blotting using standard techniques. Samples were electrophoresed on SDS-PAGE gradient gels 4–20% (TGX stain free, BioRad) and proteins transferred onto nitrocellulose membranes (BioRad). Membranes were blocked during 30 min with PBST-M (10 mM phosphate, 2.7 mM potassium chloride, 137 mM sodium chloride, pH 7.4 0.1% tween 20 (Sigma), 5% milk) and incubated with dilutions 1:1000 of anti-FLAG (Sigma, F1804 1 μg/μl) overnight at 4°C (or 2 h room temperature). Antibody binding was detected using the respective anti-mouseHRP secondary antibody (Proteintech) and Clarity ECL Western Blotting Substrate for chemiluminescence in a ChemiDocMP imaging system (BioRad).

#### UV crosslinking

For each strain, 100 ml bacterial cultures were grown to an OD_600_ of 2.0. Cultures were either directly irradiated in LB (no centrifugation step before irradiation) by placing on Petri dishes which were kept on ice, exposure to UV light (λ = 254 nm) at 5 J/cm^2^ in a CL-1000 ultraviolet crosslinker device (Ultra-Violet Products Ltd) and centrifuged at 4°C for 10 min or centrifuged at room temperature for 10 min at 15 000 g and the pellets resuspended in 0.1 vol. of the original volume with water irradiated, and then centrifuged again (note that LB medium strongly absorbs UV light at 254 nm wavelength, resulting in inferior cross-linking efficiency).

#### Immunoprecipitation and PNK assay

Immunoprecipitation and PNK assay of bacterial FLAG-tagged proteins and radioactive labeling of RNA by PNK was performed as described (14). For details see Supplementary Methods Paragraph 1.5.

## RESULTS

### TriPepSVM accurately recovers known RBPs with few false positives

We propose TriPepSVM, a SVM-based model to discriminate RNA-binding proteins from non-RNA binders based on the amino acid sequence of the protein of interest. We apply it to the proteomes of human, *Salmonella* and *E. coli*.

After collection of the data (see Paragraph *Data Sets*), we compute the best combination of hyper-parameters *k* (*k*-mer length), *C* (model cost) and *W*_+_ and *W*_−_ (class weights) during model training by conducting a grid search in a 10-fold cross-validation setting (see Paragraph *Model training*). We select *C* = 1 and *k* = 3 for all three taxa, as this combination always yielded the best balanced accuracy during the hyper-parameter tuning. We further identify the optimal class weights for human and *Salmonella* of *W*_+_ = 1.8 and *W*_−_ = 0.2. For *E. coli*, we identify *W*_+_ = 1.0 and *W*_−_ = 0.1.

**Figure 2.**
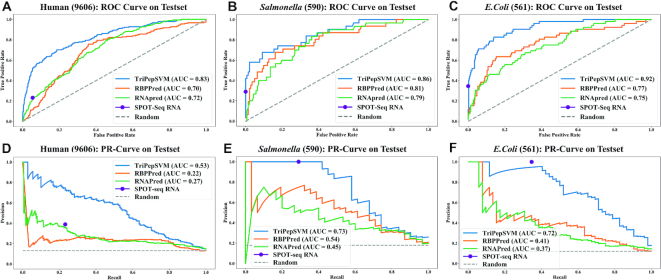
Performance of TriPepSVM in comparison to other RBP prediction methods. We compared our method TriPepSVM to RNAPred, RBPPred and SPOT-seq-RNA on human, *Salmonella* and *E. coli* test sets. Each column shows the ROC and PR curves of one organism (**A** & **D** for human, **B** & **E** for *Salmonella* and **C** & **F** for *E. coli*). SPOT-seq-RNA only outputs a class and no probability or score associated with the predictions and is hence represented only as a dot in the PR/ROC curves.

With the optimal combination of parameters at hand, we evaluated TriPepSVM on a held-out test set, which was neither used for training, nor hyper-parameter tuning (see paragraph *Model training*). We show that TriPepSVM is capable of recovering most known RBPs in the test set while still maintaining a good specificity (few false positives), yielding an area under the ROC curve (AUROC) of 0.83 and an Area under the Precision Recall Curve (AUPR) of 0.53 in human (see Figure 2). We compared the performance of TriPepSVM with formerly introduced methods for RBP prediction, namely SPOT-seq-RNA, RNApred and RBPPred and show that TriPepSVM outperforms all competing methods by a considerable margin in human (see Figure 2). SPOT-seq-RNA outputs a single classification result and no confidence score for it, hence its performance reduces to a single point on both of the curves.

We further investigated if the class-weight SVM performs similarly to a down-sampling approach and found that both approaches give similar results on the held-out test set (Supplementary Figure S5).

Performance measures for all tools computed at the optimal PR curve cutoff (reported in Table 2 and determined as described in Supplementary Paragraph 1.4) are given in Supplementary Figure S4, for human, *E. coli* and *Salmonella*. TriPepSVM outperformed all other methods in terms of MCC on all three species and had a slightly higher balanced accuracy compared to the best and most recent tool RBPPred. In addition, TriPepSVM reaches a good compromise between sensitivity and specificity at the optimal cutoff (Supplementary Figure S4). In comparison, SPOT-Seq-RNA has a high specificity, especially for *E. coli* (Figure 2 F) but low sensitivity, i.e. it misses a large amount of known RNA-binders. On the other hand, RNAPred exhibits a high sensitivity, relying only on amino acid composition, at the expenses of a poor specificity. The optimal cutoffs determined by TriPepSVM on human, *Salmonella* and *E. coli* were used to carry on proteome-wide predictions on the three species.

**Table 2.** Optimal classification cutoff for the tested prediction tools and organisms

Application	Human	*Salmonella*	*E. coli*
TriPepSVM	0.68	0.28	0.3
RBPPred	0.81	0.34	0.35
RNAPred	−0.24	−0.18	−0.84

### Runtime comparison

We measured the runtime in predicting RBPs on sequence sets of increasing size (Supplementary Figure S7). Equally to RNApred, TriPepSVM exhibited the best performance, with a constant runtime in the order of seconds on a single CPU. On the other hand, the runtime of RBPPred and SPOT-seq-RNA increased linearly with the size of the data sets, up to 83 hours and 12 days for 250 sequences, respectively (Supplementary Figure S7). This highlights that structure template-based methods do not scale sufficiently well when applied in a proteome-wide fashion.

### TriPepSVM results are consistent with interactome capture studies

**Figure 3.**
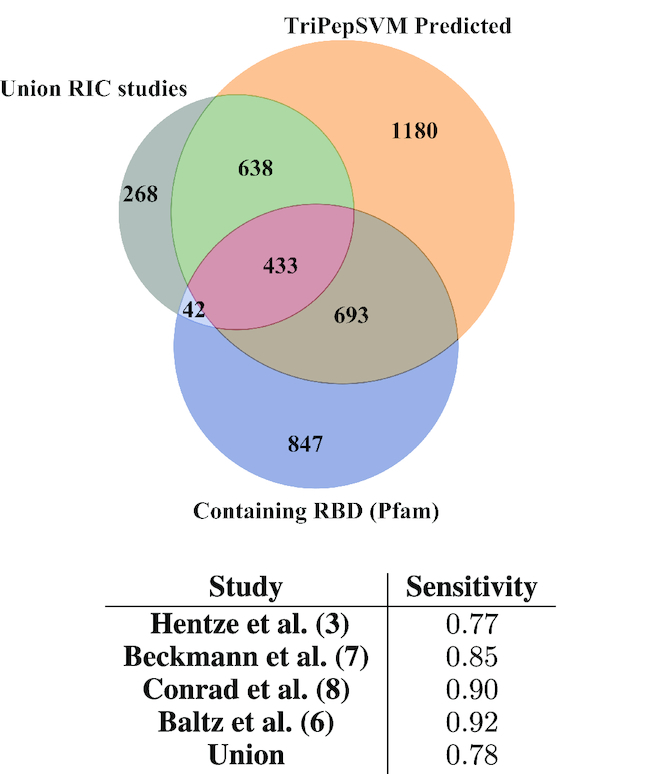
Overlap between four different interactome capture studies and predictions from TriPepSVM.We computed the overlap between our proteome-wide predictions (orange, top right), the union of identified proteins from four independent RIC studies (gray, top left) and proteins containing RBDs according to the Pfam database (blue, bottom). The table shows the sensitivity between our predictions and the four different RIC studies and their union.

We found 2944 proteins with a predicted RNA-binding capability in the human proteome (see Supplementary Table S13). To assess whether these proteins are really likely to bind RNA, we overlapped our predictions with interactome capture studies from recent years. Figure 3 shows the overlap between our predictions (TripPepSVM Predicted), the union of discovered RBPs by four different interactome capture studies and proteins that contain a Pfam RBD. The table in Figure 3 reports the fraction of proteins from the interactome capture studies which our model was able to recover for the tuned cutoff of 0.68. For all of the four studies, we were able to recover >75% of the experimentally identified RBPs, and for three of these studies this percentage was higher or equal to 85%.

### TriPepSVM predicts novel human RBPs

It should be taken into account that the vast majority of RIC-RBPs are mRNA-binders since they were identified using poly-A RNA selection and eukaryotic mRNA only constitutes for a small subset of cellular RNA (around 5%). TriPepSVM, however, was trained on a superset of all known RBPs. Consistent with this, we also identify 14 cytosolic and 6 mitochondrial tRNA ligases, 6 tRNA/rRNA methyltransferases (NSUN2-6) and the majority of ribosomal proteins (31 out of 33 small subunit proteins and 44 out of 47 large subunit proteins (41)).

The recent consensus for human RBPs ranges from hundreds (1) to >2000 (3) RBPs. From the 2944 proteins predicted by TriPepSVM, 990 (34%) were not previously described in the aforementioned RIC studies, do not contain a known RNA-binding domain or were not annotated as RBPs.

Among the 990 predicted RNA-binders are >200 proteins with ATP-binding capacity, from these are 13 proteins of the AAA ATPase family. The overlap between RNA- and single nucleotide binding is generally very high among the established RBPs as well (8). Also enriched are kinases, proteins harboring WD40 domains and bromodomain folds. During the writing of this manuscript, we and others published work in which RBPs were identified by biochemical techniques (called PTex (42) and XRNAX (43)) in a poly-A independent fashion. The findings in these studies are largely confirmatory to our results as proteins of the AAA ATPase family, WD40 and bromodomain proteins were likewise identified as novel RBPs.

### Important sequence patterns in RBPs and their biological significance

We next set out to identify those k-mers that contributed the most to classifying RBPs versus non-RBPs in human, *E. coli* and *Salmonella*, using Equation 2 (see Paragraph *Feature importance scores*). The highest ranked *k*-mers (top 50 ranking) are listed in the Supplementary Tables S7–S9 for human, *Salmonella* and *E. coli*, while in Supplementary Tables S10–S12 all *k*-mers with their corresponding weights are reported. In all three organisms, *k*-mers containing lysine (K), arginine (R) and glycine (G) are found to have the largest SVM weight. Finding K and R enriched among the triplets was expected since positively charged residues are known to be involved in direct RNA interaction; e.g. the RGG box is a known RNA-binding motif in unstructured regions of proteins (11). Although an aromatic motif YGG has been identified as RNA-binding site by Castello and colleagues (11), in our dataset YGG is not among the top k-mers which contribute to RBP classification with a weight close to 0. YGG repeats (also called [G/S]Y[G/S] motif) can bind to RNA and promote hydrogel formation *in vitro* as well as liquid-liquid phase separations (LLPS) *in vivo* (44,45). GYG, SYS, GYS and SYG triplets, which are potentially part of the [G/S]Y[G/S] motif, although not among the top 50 important k-mers in our human model, have all positive weights, indicating that they contribute to the recognition of the RBP class. Nearly all *k*-mers with a negative weight (contributing to classification as non-RBP) contain leucine (L) and/or glutamic acid (E). Consistent with this, E and L are the residues most absent from RNA-binding sites in human cells (11). In particular, the *k*-mer LLL is depleted in the RBP class of all three organisms (Supplementary Tables S7–S9).

**Figure 4.**
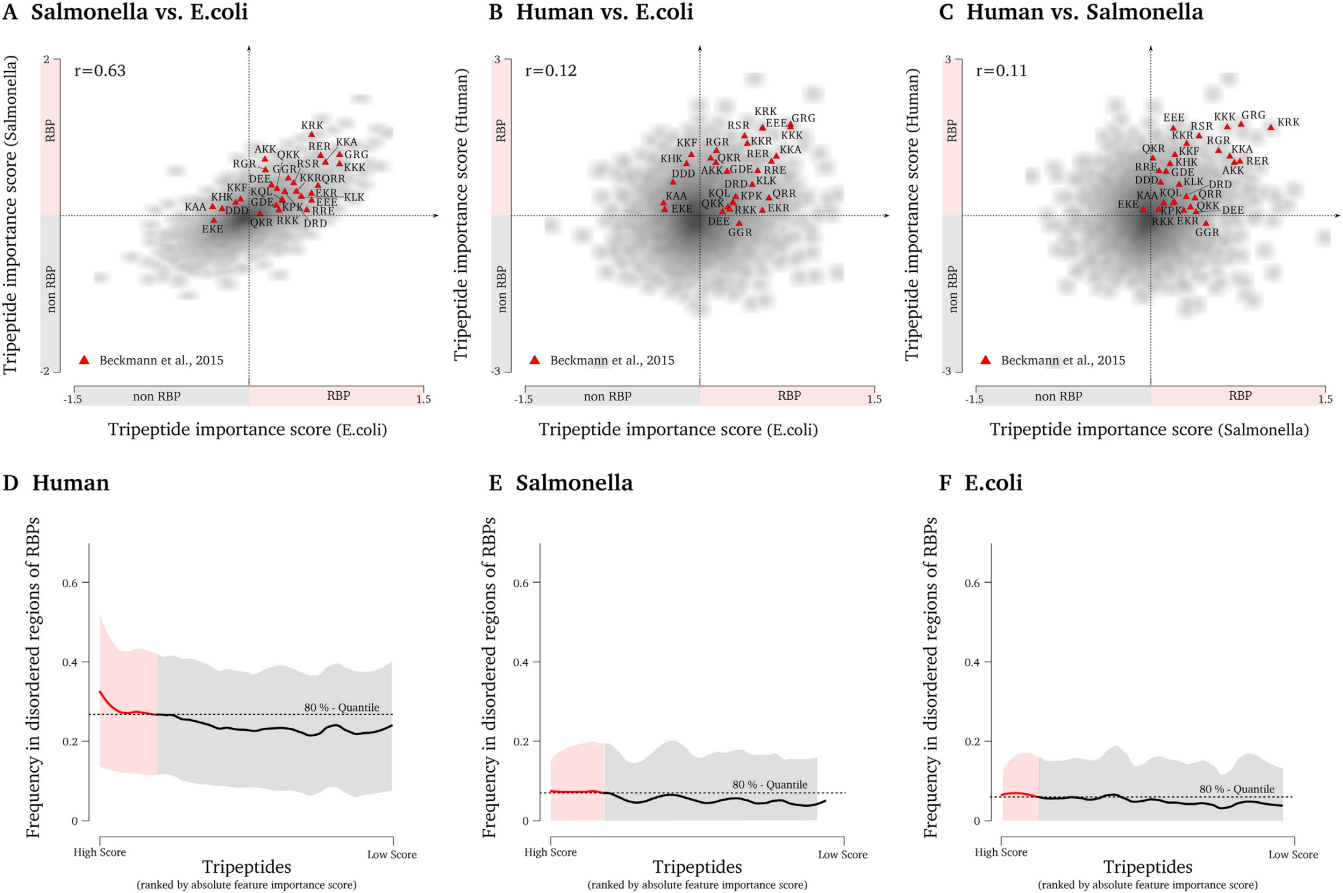
TriPepSVM classifiers apply highly conserved tripeptides that are enriched in structurally disordered regions of RBPs in human. **A, B, C** show tripeptides (red triangles) identified by Beckmann *et al.* (7) as conserved in eukaryotic RBPs and expanded from yeast to human with their corresponding weights for A *Salmonella* vs. *E. coli*, B human vs. *E. coli* and C human vs. *Salmonella*. Most of the conserved tripeptides from (7) harbor positive weights not only in human but also in *Salmonella* and *E. coli*, and are therefore important in characterizing RNA-binders in human and bacteria. We report pearson’s correlation coefficient for all pairwise comparisons and show high correlation (*r* = 0.63) between the feature weights extracted from the classifiers trained on bacteria. **D, E, F** show the structural disorder fraction of each tripeptide ranked by the absolute feature importance score. The resulting curve is smoothed using LOESS regression (span = 0.2) where the shading shows the standard deviation. The dashed line marks the value of the 80%-quantile of the smooth LOESS regression values. The frequency indicating how often a tripeptide is observed in the structural disordered part of an RBP increases with the feature importance score in D human, but not in E *Salmonella* and F *E. coli*.

To investigate whether the rules learned by the three classifiers are universal or unique to the organism which it was trained on, we inspected the top k-mer features learned by TriPepSVM for human, *Salmonella* and *E. coli* (Supplementary Table S7–S9). As expected, the pairwise correlation between all learned *k*-mer weights for two organisms is much higher for *Salmonella* and *E. coli* (Pearson correlation of 0.63) than between human and *Salmonella* (Pearson correlation of 0.11) or human and *E. coli* (Pearson correlation of 0.12) (Figure 4A–C).

Next, we compared the weights of all *k*-mers from human, *E. coli* and *Salmonella* to our previously identified triplets conserved in eukaryotic evolution (7) (see red triangles in Figure 4A–C). Our findings independently confirm that those triplets are not only conserved among RBPs but are also important to correctly identify these proteins as RNA-binders by TriPepSVM, given that most of them have a positive weight (Figure 4A–C). Interestingly, a high portion of *k*-mers found to be conserved in eukaryotic RBPs (mainly KR- and RG-containing triplets) seem to be important not only for human, but also for bacterial RBP classification.

Following on the presence of *k*-mers which are known to bind RNA in unstructured regions, including those containing G/Y and R/G, we probed for all three species whether overall the top *k*-mers identified by our SVM model had a higher propensity to be found in structured domains or unstructured parts of proteins (see Figure 4D–F) using IUPred prediction (38). Strikingly, k-mers with the biggest contribution to classify a protein are more often found in disordered regions in human, but not in *Salmonella* and *E. coli*. This indicates that different properties are encoded in the sequence features of eukaryotic versus bacterial RBPs. In addition, we predict known human RBPs, such as HNRPU, PTBP1, FUS, SRSF1, U2AF2 and DDX4, that are known to mediate RNA binding via disordered regions and are implicated in several aspects of RNA processing (see Supplementary Table S13).

### Searching a bacterial proteome for RBPs

We next used TriPepSVM to predict potential RBPs in a prokaryotic organism. As for most bacteria, RBPs are poorly annotated in *Salmonella* Typhimurium despite recent advances such as the identification of the Csp proteins or ProQ as RNA-binders by Smirnov and colleagues. The same study, however, indicated that more, so far un-identified proteins harbor the potential to bind RNA (46). After training TriPepSVM on bacteria from the *Salmonella* clade (using the recursive mode for UniprotKB taxon 590) to obtain a larger positive training set, we searched the complete *Salmonella* Typhimurium proteome for RBPs. We correctly identify 108 of the Uniprot-annotated 115 RBPs, resulting in a 94% recovery rate. We correctly predict Hfq (14), and ProQ (46) which are bacterial mRNA- and sRNA-binding proteins. Recently, cold schock proteins CspC and CspE were described as RNA-binding proteins in bacteria (47) and *Salmonella* (48). Unfortunately, cspE is still not present in the reviewed section of SwissProt and therefore, we did not include it in our proteome data (see Paragraph *Methods*). CspC, on the other hand, is present in SwissProt but was not yet marked as RNA-binding and we fail to predict it for *Salmonella*. In *E. coli*, however, we correctly predict CspC as RNA-binder.

Using the tuned cutoff of 0.28 for *Salmonella* (see Table 2), we additionally predict 66 additional proteins to bind to RNA (see Supplementary Table S14). Among those are 8 ribosomal proteins and 14 other proteins involved in RNA biology all of which are not annotated as ‘RNA-binding’ such as the GTPase Der which is involved in ribosome biogenesis (49), ribosomal methyltransferases RimO and RlmE (50), RNA pyrophosphohydrolase RppH (51), or transcription elongation factors GreA/B (52). Furthermore, we predict 20 known DNA-binding proteins and 18 proteins with documented ATP-binding activity to be RNA-interacting. Additionally, 18 predicted proteins are not implicated to interact with RNA or any other nucleic acid; 12 of those have enzymatic activity, consistent with a growing list of enzymes from diverse species to be associated with RNA (7,10,53).

### Experimental validation of predicted RBPs in *Salmonella*

**Figure 5.**
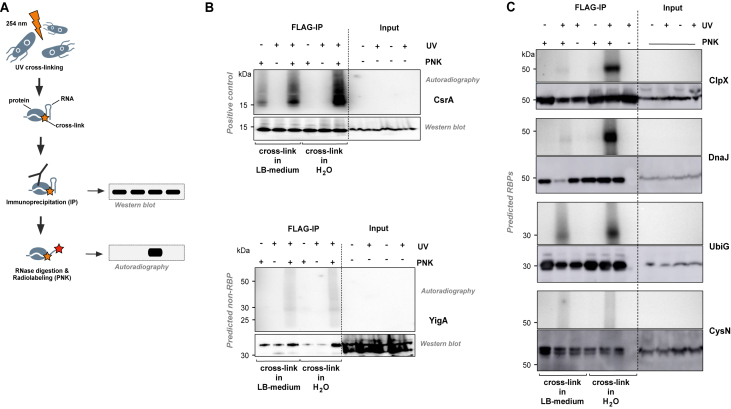
Experimental validation of predicted bacterial RBPs. (**A**) Schematic view of the PNK assay. After UV cross-linking of ribonucleoprotein complexes *in vivo*, cells were lysed. UV irradiation can result in a covalent link (orange star) between RNA and proteins that are in close physical proximity (zero distance cross-link). Individual candidate RBP-FLAG fusions were then immunoprecipitated (IP) with a FLAG-specific antibody. After trimming of the co-imunoprecipitated RNA using RNase digestion, polynucleotide kinase (PNK) was used to enzymatically add a radioactive phosphate (^32^P) to 5’-ends of transcripts. Finally, input controls as well as the IPs were separated by SDS-PAGE. After blotting to a membrane, protein amounts were analysed by Western blotting; presence of a radioactive signal at the same molecular mass as the protein then serves as indirect proof of RNA-binding. (**B**) PNK assay of CsrA (positive control) and YigA (predicted non-RBP). UV cross-linking was performed in LB medium and water independently. A radioactive signal can only be detected for RBPs (here: CsrA) after UV cross-linking and in presence of PNK. (**C**) PNK assay of four candidate RNA-binding proteins in *Salmonella* Typhimurium predicted by TriPepSVM. ClpX, DnaJ and UbiG could be confirmed to interact with RNA *in vivo*, but not CysN.

Next, we set out to experimentally validate TriPepSVM predictions *in vivo*. We generated *Salmonella* mutant strains carrying FLAG-tagged RBP fusion in its genomic context using the λ Red method; resulting in bacterial mutants that exclusively express the predicted FLAG-tagged RBP candidate at their respective physiological levels (40). We chose ClpX (a subunit of the Clp protease regulating expression of the flagellum (54)), DnaJ (a chaperone responding to hyperosmotic and heat shock (55)), UbiG (a ubiquinone biosynthesis O-methyltransferase (56)) and CysN (Sulfate adenylyltransferase subunit 1 (57)) as predicted RBPs and YigA which TriPepSVM predicts as non-RBP and we tested for RNA-binding *in vivo* using the PNK assay (see Figure 5A). As demonstrated by a radioactive signal from 5’ end labeled co-immunoprecipitated RNA (see Figure 5B, C), we can confirm that we correctly predicted ClpX, DnaJ, UbiG (RNA-binding) and YigA (true negative) but not the candidate CysN which was also not predicted as RBP by the human or *E. coli* models. The validation of four out of five proteins is therefore also matching with our calculated balanced accuracy of 73% (see Supplementary Figure S4). Importantly, ClpX has ATP-binding activity and DnaJ is able to bind to DNA and ATP (54,55). To exclude self-phosphorylation or direct binding from ATP-binders to the radioactive isotope in our assay (58), we also included conditions in which PNK was omitted but P^32^-γ-ATP was provided (see Figure 5B, C). However, our validation demonstrates that both are bona-fide RNA-interactors *in vivo* from which we conclude that TriPepSVM is unlikely to incorrectly predict DNA- or single nucleotide binders as RBPs.

### Cross-species predictions

Finally, we investigated how well a classifier trained on one organism can predict RBPs in another organism. This is particularly useful because it potentially allows us to improve RBP annotation in species whose genomic sequence is known but RBP annotation is scarce, as it is the case for many bacterial genomes. As expected and shown in Supplementary Figure S7, TriPepSVM achieves the best BACC when training and testing on the same species. In general, *Salmonella* RBPs are well predicted by TriPepSVM trained on *E. coli* and viceversa (BACC of 80% in both cases), in line with the observation that the sequence *k*-mers required to identify RBPs are very similar in both organisms (Figure 4A). However, a human classifier, where sequence features are more complex and related to structurally disordered regions (Figure 4D), performs less well in cross-species prediction when tested on both *E. coli* and *Salmonella* (Supplementary Figure S7).

In a second step, we used TriPepSVM trained separately on *Salmonella, E. coli* and human to assess how they behave proteome-wide in cross-species RBP prediction. When applied to the whole human proteome, the human model predicts 2944 RBPs, but only 38% and 40% of those are predicted by the *E. coli* and *Salmonella* model as well (Supplementary Figure S8A). This is best exemplified by the Pumilio proteins PUM1 and PUM2, both members of the PUF protein family and highly conserved from human to fly (59). They are correctly predicted as RBP by the human-trained model but not by bacterial-trained TriPepSVM. Next to the RNA-interacting Pumilio-homology domain (Pum-HD) both human proteins contain a large N-terminal unstructured part (59). PUM3 however, which contains the Pum-HD but lacks the disordered region of PUM1 and PUM2, is correctly identified by the bacterial models as well. On the contrary, on the *Salmonella* proteome the *E. coli* and the *Salmonella* models exhibit a large overlap and can predict up to 84% of common RBPs, while the human classifier can only predict 34% of those (Supplementary Figure S8B). Similar results are found when applying all three models on the *E. coli* proteome, with the *E. coli* and *Salmonella* models having 74% of common RBP predictions (Supplementary Figure S8C). Here, the human-trained model identifies conserved RBPs such as several ribosomal proteins but fails to correctly predict the bacterial RBPs Hfq, ProQ and CsrA. In conclusion the overlap between the predictions of the two bacterial models is much higher than with the human model in all cross-species comparisons. This is expected given the low correlation between the learnt sequence features from the human and the bacterial models versus the high correlation of learned features between *E. coli* and *Salmonella* models. The results clearly show that cross-species prediction works best across evolutionary-related species.

## DISCUSSION

What defines a RNA-binding protein? Apart from the obvious functionality to bind to RNA, other elements within a protein can be important to exert its physiological role in the cell. With TriPepSVM, we are presenting an approach which reduces a protein to its combination of short amino acid k-mers, in our case triplets, and use machine learning to find patterns in these combinations that align with RNA-binding. *Escherichia coli* is a well-studied gram-negative bacterium of the intestinal microflora and used as model organism in several studies. Its genome and proteome have been extensively annotated, providing therefore also hundreds of annotated RBPs for training predictive models. *Salmonella* Typhimurium is also a well-studied bacterium; not the least due to its role as Gram-negative model organism to study infection by prokaryotic pathogens. Despite its importance, only a very limited set of RNA-interacting proteins has been identified in *Salmonella* and other bacteria beyond the canonical set of proteins which make up the transcription machinery, the ribosome or interact with tRNA. In recent years, novel approaches such as Grad-Seq (46) identified additional proteins that can interact with bacterial mRNA. So far, 5 mRNA-binding proteins have been confirmed in *Salmonella*: Hfq, CsrA, ProQ and CspC/E. This limited data set renders prediction and discovery of novel RBPs in bacteria a challenging task since most bioinformatic prediction tools depend on either structural similarity to protein folds that are known to be involved in RNA interaction or on homology based on phylogeny. In both cases, the limited availability of known bacterial RBPs represents an important obstacle - also for our method. Still, our approach correctly identifies most known RNA-binders and predicts 66 novel candidate RBPs in *Salmonella* from which we tested ClpX, DnaJ, UbiG and CysN for validation. Indeed, three (ClpX, DnaJ and UbiG) out of the four could be confirmed to bind RNA *in vivo* (see Figure 5).

In our approach, we reduce the search space to the most basic feature of any protein: its primary sequence. Following the observation that i) many recently-found RBPs lack known RNA-binding domains (11) and ii) our earlier work showed an expansion of short triplet amino acid motifs in RBPs throughout evolution (7), TriPepSVM rather searches for combinations of triplet peptides in proteins then for full domains. This reduction in complexity has the advantage that TriPepSVM is independent on prior (and potentially biased) knowledge on RBDs or homology. We demonstrate that highly informative sequence features are contained within RBPs of both, human and the two bacteria *Salmonella* and *E. coli*. Using annotated RBPs for both training and evaluation of our method, we are able to show that protein sequences can be confidently used as a predictor for RBPs in all three species.

Additionally, we have shown that RBP predictions made by training a classifier in one species and predicting in another can be very accurate when performed between related species, while cross-species prediction is more difficult between human and bacteria. This can be explained by the different rules learned by classifiers trained on different species, with *Salmonella* and *E. coli* sharing many more common sequence *k*-mers in their RNA-binding proteome than human and *Salmonella* or human and *E. coli*.

The fact that TriPepSVM does not classify all human proteins with a Pfam-domain (see Figure 3) as RBP also demonstrates that tripeptides from RNA-binding domains alone are not sufficient to explain the performance of TriPepSVM. Intriguingly, tripeptides which we predict to contribute to RNA-interaction more prominently have a tendency to be enriched in structurally disordered regions in human but not in *E. coli* and *Salmonella* (see Figure 4D–F). Together with our earlier comparison of tripeptide motifs in eukaryotic RBPs in which unicellular yeast harbors few tripeptide repetitions that expand during evolution, it is tempting to speculate that RNA-binding via unstructured regions is of higher physiological relevance in more complex organisms than in unicellular species. Consistent with this hypothesis is the observation that sequence-independent RNA-binding in unstructured regions of RBPs is important for P-bodies or RNA granules by liquid-liquid phase transitions (60). Formation of these higher-order RNA-protein complexes, however, has not been described for bacteria so far. Our results show that if present in prokaryotes, regulation of RNA-granule-like complexes is very unlikely through unstructured regions of RBPs.

## CONCLUSION

All in all, we show that the propensity of a protein to bind RNA is mostly encoded in its primary sequence and can be confidently predicted based solely on combinations of short amino acid triplets. TriPepSVM outperforms previous approaches which make use of more complex protein features in discriminating RBPs from non-RBPs. It can in principle be applied to any species, from eukaryotes to bacteria where limited experimental data are available. Besides being a valuable RBP prediction method from sequence alone, our approach can pinpoint the important sequence patterns which distinguish RBPs from non-RBPs and points to disordered regions as main determinants of RBP-RNA interactions, in line with the latest studies.

## DATA AVAILABILITY

The collection pipeline as well as the source code for TriPepSVM are available on Github, https://github.com/marsicoLab/TriPepSVM.

## Supplementary Material

Supplementary Data
