# Supplementary material for: TriPepSVM: de novo prediction of RNA-binding proteins based on short amino acid motifs
Source: Nucleic Acids Res. 2019 Mar 29;47(9):4406–17. doi: 10.1093/nar/gkz203 (PMC6511874; doi:10.1093/nar/gkz203)
Supplement: Supplementary Data [file gkz203_supplemental_files.zip › Supplementary_Data.pdf]

Supplementary Materials

1 Methods

1.1 Data Collection

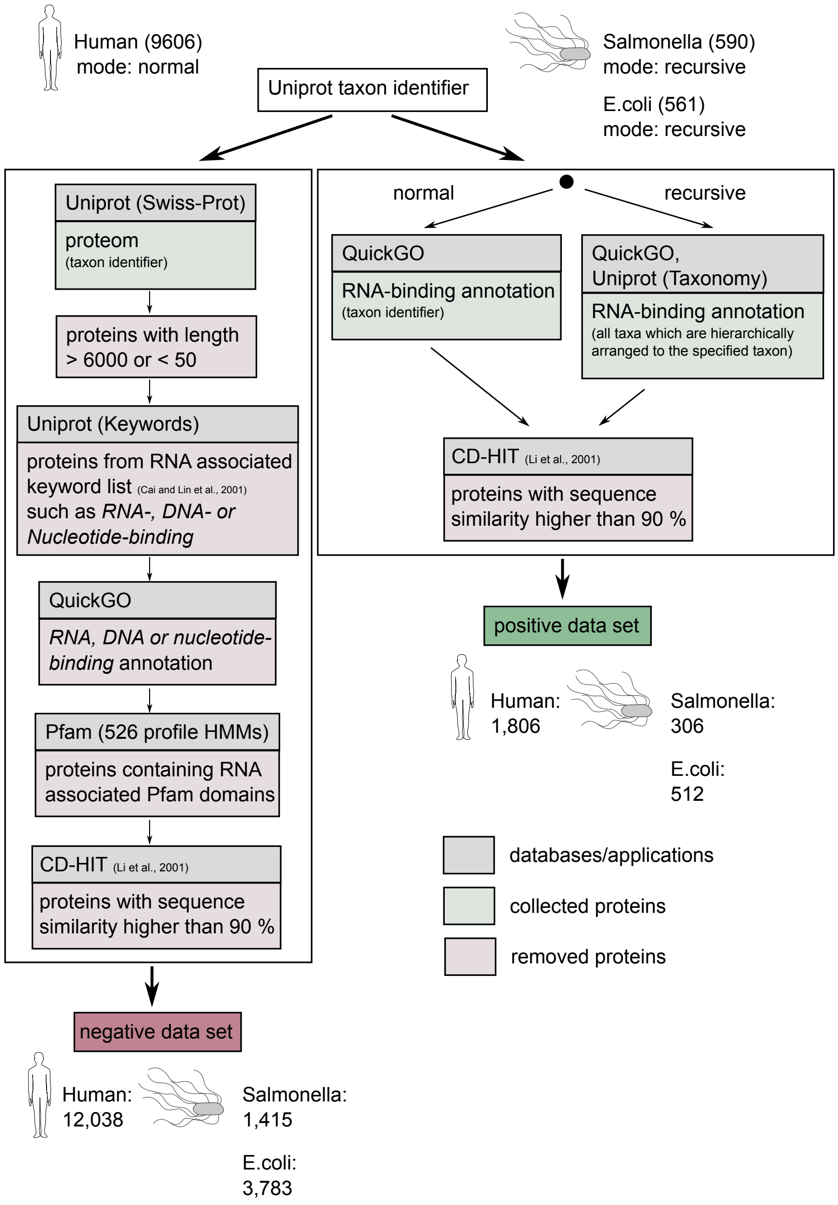

Figure S1: **Pipeline to collect RBPs (positive data) and non-RBPs (negative data).** The fully automated pipeline requires the Uniprot identification number of a taxon to collect positive and negative data sets. The data sets form the foundation to train and validate the TriPepSVM classifier. The pipeline supports a recursive mode to collect positive data from all members of the specified taxon. In the figure we see the results for human (9606, normal mode), Salmonella (590, recursive mode) and E.coli (561, recursive mode). Light red boxes in the workflow correspond to filtering operations on the input sequences, while green boxes correspond to collection steps on the input sequences.

1.2 Parameter Tuning

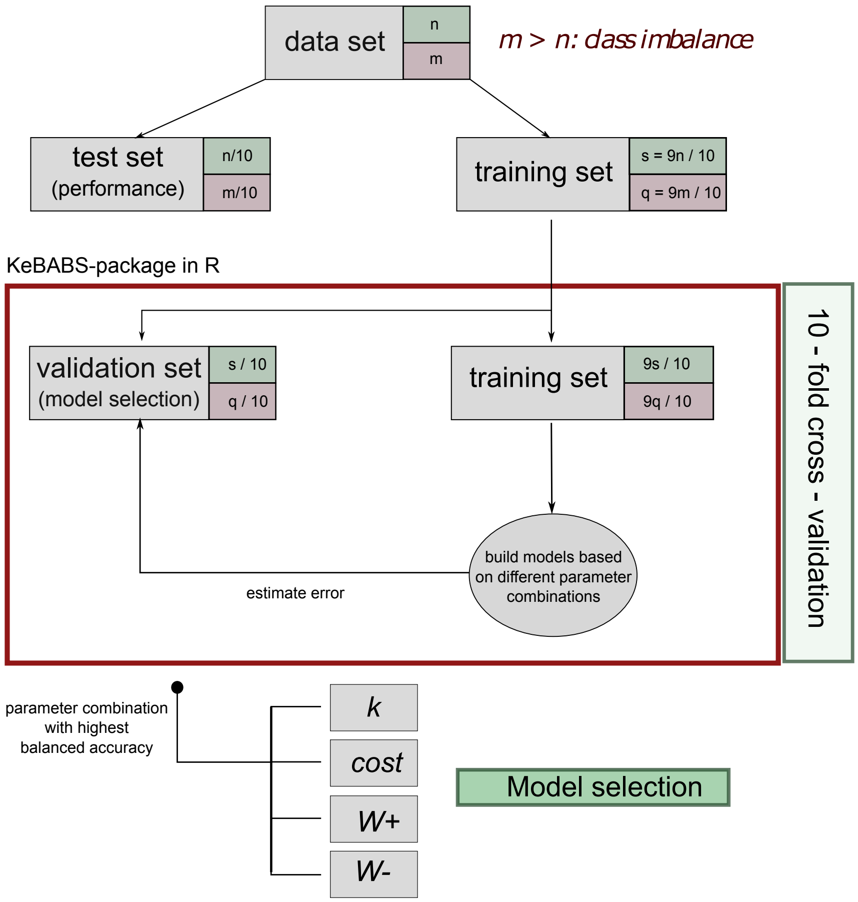

Figure S2: **Data splitting and parameter tuning.** The collected positive and negative proteins are pictured as data sets with n positive and m negative elements. Those collected data sets present class imbalance, containing much more negative than positive examples. Firstly, we split the data set into training (90 %) and test set(10 %). We apply the *KeBABS R* package to perform a 10-fold cross-validation to identify parameters k and *cost*. In detail, we split the data into $n = 10$ equally sized subsets and in each of the n iterations $n - 1$ splits are used for training and the i split is used as validation to compute the classifier performance. Subsequently, the parameter combination resulting in the smallest average balanced accuracy is selected. Since the training of the class weights, denoted with $W+$ and $W-$, is not supported in the *KeBABS*-package, we run a separate outer loop for the selection of a good combination of weights for the positive and negative class. The final classifier is trained using the combination of hyper parameters that achieves maximum average balanced accuracy.

1.3 Performance Metrics

To evaluate the performance of our classifier and the other RBP prediction methods we computed the True Positives (TP) as the correctly identified RBPs, the False Positives (FP) as those proteins misclassified as RPBs, the True Negatives (TN) as those proteins which are correctly identified as

non-RBPs and the False Negatives (FN) as those undetected RBPs. These values are then used to compute several performance metrics in order to compare TriPepSVM to previous approaches. We mainly compute the area under the precision-recall curve (AUPR) and the area under the receiver operating characteristic curve (AUROC) (figure 2 in the main article). In addition, we computed the balanced accuracy (BACC) to account for class imbalance, sensitivity, specificity and matthews correlation coefficient (MCC). AUPR, AUROC, BACC and MCC are explained more in detail below.

1.3.1 Area under the Precision-Recall Curve (AUPR)

The area under the precision-recall curve is a classifier's performance metric independent from the chosen cutoff to classify examples in the positive or the negative class. In particular, when a classifier outputs a probability or any other continuous value (for instance the distance of a vector from the decision boundary of an SVM), one can compute pairs of precision and recall for all possible cutoffs. This function of the cutoff can be plotted and is commonly referred to as the precision-recall curve (PR-curve). The best possible PR-curve goes from the upper left corner (perfect precision with no recall) straight to the upper right corner (perfect precision with perfect recall), and then it falls to the bottom right corner (perfect recall with no precision). Such an ideal PR-curve has an area under the curve of 1. A random classifier has a AUPR given by the fraction of positives examples in the dataset.
PR-curves have also the advantage to account for class imbalance. In fact, a classifier that appears to perform well in the balanced setting might be nearly useless in the unbalanced setting, because it is very hard to control the number of false positives in such a situation. To circumvent this, PR-curves plot the fraction of predicted example (in this case RBPs) that are true (precision) versus the fraction of all true RBPs that are predicted as RPBs (recall) at different cutoff scores. In our case they would give a good idea of whether the proteome-wide predictions of each classifier are likely to contain many false positives.

1.3.2 Area under the receiver operating characteristic curve (AUROC)

Similar to the precision-recall curve, it is possible to investigate the true positive rate (TPR), given by the number of true positives over the total number of positives, and the false positive rate (FPR), given by the number of false positives over the total number of negatives, as a function of all thresholds. This is referred to as the receiver operating characteristic curve (ROC curve), where the FPR is on the x-axis and the TPR on the y-axis. The ideal curve goes from the lower left corner (no false positives and no true positives) straight to the upper left corner (no false positives but perfect true positives) and then to the upper right corner. Similar to the PR curve, the area under the perfect ROC curve is 1. A random guess at a certain cutoff score would give a point along the diagonal line and therefore the AUROC of a random classifier is 0.5. ROC curves have the advantage to be independent of a specific cutoff but do not account for class imbalance. ROC curves are known to be insensititve to class imbalance, i.e. the ratio of RBPs versus non-RBPs in the genome, making it hard to control the number of false positives.

1.3.3 Balanced Accuracy

Balanced accuracy (BACC) is a special form of accuracy which tries to account for a case where the classes are not balanced in a dataset. BACC is given by the equation:

$$BACC = \frac{\left(\frac{TP}{P} + \frac{TN}{N}\right)}{2} \quad (1)$$

Hence, BACC is essentially the mean of TPR and specificity and it is dependent on the chosen cutoff score to classify examples in the positive or the negative class.

1.3.4 Matthews Correlation Coefficient (MCC)

The matthews correlation coefficient computes the correlation coefficient between the true labels and the observed classification results. As the correlation coefficient, it returns a value between -1 and 1 with 0 corresponding to no correlation at all (random performance).
MCC is suited for situations in which the classes are not balanced but it depends on choosing a specific cutoff. MCC is defined as:

$$MCC = \frac{TP \times TN - FP \times FN}{\sqrt{(TP + FP)(TP + FN)(TN + FP)(TN + FN}}. \tag{2}$$

1.4 Choosing Optimal Cutoff Values

In order to enable a fair comparison between all four tools and to be consistent with our proteome-wide predictions, we compute optimal cutoff values from the PR-curves of all classifiers except SPOT-seq RNA. We obtain the optimal cutoff by computing the point on the PR curve that is closest to the optimal curve in euclidean space. For a given cutoff t, we define the resulting precision and recall of a classifier under t as $pr_c(t)$ and $rec_c(t)$, respectively. We can then define the optimal cutoff for a classifier c as:

$$\hat{T_c} = arg \min_t \left(\sqrt{(1 - rec_c(t))^2 + (1 - pr_c(t))^2} \right) \tag{3}$$

To enable a fair comparison, we compute this optimal cutoff for all compared methods to obtain the results of figure S4.

1.5 Cell Culture & Molecular Biology

1.5.1 Construction of bacterial strains

To tag candidate RBPs, a x3FLAG::KmR cassette was inserted into the genomic loci of the respective genes of *Salmonella* Typhimurium SL1344. The gene::x3FLAG::KmR was constructed following the procedure based on the Lambda Red system developed by Datsenko and Wanner. The system is based on two plasmids: pKD46, a temperature-sensitive plasmid that carries gamma, beta and exo genes (the bacteriophage λ red genes) under the control of an Arabinose-inducible promoter, and pSUB11, carrying the x3FLAG::Km^{R} cassette. The cassette in pSUB11 was PCR-amplified with primers (see Table S3), the 5 ends of which were designed to target the 3end of the gene of interest (C-terminal tag), digested with DpnI at 37°C for one hour and, upon purification, used for subsequent electroporation. *Salmonella* Typhimurium SL1344 harbouring plasmid pKD46 was grown in LB containing Ampicillin (100 μg/ml) and L-Arabinose (100 mM) at 30°C to an OD_{600} of 0.8. Cells were incubated on ice for 15 min, and centrifuged for 30 min at 3220 x g at 4°C and resuspended in ice-cold water. The wash was repeated three times. On the final wash, cells were resuspended in 300 μl water and electroporated with 200 ng of PCR product. Cells were recovered for one hour in LB at 37°C on a tabletop thermomixer at 600 rpm, plated on LB agar with Kanamycin (50 μg/ml) overnight. The following day, 10 colonies per strain were picked, resuspended in PBS and streaked on plates containing Ampicillin or Kanamycin and incubated at 40°C. Colonies that showed resistance to Kanamycin but not to Ampicillin were selected for further analysis, and the correct expression of the epitope tag was verified by western blot.

1.5.2 Immunoprecipitation

Cell pellets were resuspended in 800 0.2μl NP-T buffer (50 mM NaH_2PO_4, 300 mM NaCl, 0.05% Tween, pH 8.0) together with 1 ml glass beads (0.1 mm). Cells were lysed by shaking at 30 Hz for 15 min at 4°C and centrifuged for 15 min at 16,000 g and 4°C. Cell lysates were transferred to new tubes and centrifuged for 15 min at 16,000 g and 4°C. The cleared lysates were mixed with one volume of NP-T buffer with 8 M urea, incubated for 5 min at 65°C in a thermomixer with shaking at 900 rpm and diluted 1:10 in ice-cold NP-T buffer. Anti-FLAG magnetic beads (Sigma) were washed three times in NP-T buffer (30 μl 50% bead suspension was used for a lysate from 100 ml bacterial culture), added to the lysate, and the mixture was rotated for one hour at 4°C. Beads were collected by centrifugation at 800 g, resuspended in 1 ml NP-T buffer, transferred to new tubes, and washed 2 with high-salt buffer (50 mM NaH_2PO_4, 1 M NaCl, 0.05% Tween, pH 8.0) and 2 with NP-T buffer. Beads were resuspended in 100 μl NP-T buffer containing 1 mM $MgCl_2$ and 2.5 U benzonase nuclease (Sigma) and incubated for 10 min at 37°C in a thermomixer with shaking at 800 rpm, followed by a 2-min incubation on ice. After one wash with high-salt buffer and two washes with CIP buffer (100 mM NaCl, 50 mM TrisHCl pH 7.4, 10 mM $MgCl_2$), the beads were resuspended in 100 μl CIP buffer with 10 units of calf intestinal alkaline phosphatase (NEB) and incubated for 30 min at 37°C in a thermomixer with shaking at 800 rpm. This was followed by one wash with high- salt buffer and two washes with PNK buffer (50 mM TrisHCl pH 7.4, 10 mM $MgCl_2$, 0.1 mM spermidine).

1.5.3 PNK assay

After one wash with high-salt buffer and two washes with CIP buffer (100 mM NaCl, 50 mM TrisHCl pH 7.4, 10 mM $MgCl_2$), the beads were resuspended in 100 μl CIP buffer with 10 units of calf intestinal alkaline phosphatase (NEB) and incubated for 30 min at 37°C in a thermomixer with shaking at 800 rpm. This was followed by one wash with high-salt buffer and two washes with PNK buffer (50 mM TrisHCl pH 7.4, 10 mM $MgCl_2$, 0.1 mM spermidine). Next, beads were resuspended in 100 μl PNK buffer and 1 U T4 PNK (0.1 U/μl, NEB) and 5.5 μCi $^{32}\gamma$P-ATP were added and incubated at 37°C for 30 min. The beads were then washed twice in PNK buffer and resuspended in 50 μl of a 2x denaturing gel loading buffer (Invitrogen). 30 μl samples were then analysed on a NuPAGE Bis-Tris gel (Invitrogen) and radioactive signal was detected in a Life Science FLA-5100 imaging system (Fujifilm).

Table S1: Strains used in this study. *Salmonella enterica* subsp.*enterica* serovar Typhimurium

Strain name	Genotpye	Source
SL1344	*rpsL hisG*	Holmqvist et al. 2016
JVS-04317	*SL1344 csrA-3xFLAG KanR*	Holmqvist et al. 2016
ST-BB-2024	*SL1344 dnaJ::FLAGx3::KanR*	This study
ST-BB-2025	*SL1344 clpX::FLAGx3::KanR*	This study
ST-BB-2026	*SL1344 ubiG::FLAGx3::KanR*	This study

ST-BB-2010	*SL1344 cysN::FLAGx3::KanR*	This study
ST-BB-2004	*SL1344 yigA::FLAGx3::KanR*	This study

Table S2: Plasmids used in this study.

Name	Details	Source
pSUB11	*Km*^{R}*, 3xFLAG*	Uzzau et al. 2001
pKD46	*oriR(ColE1) repA101ts araC bla; contains* γ*,* β *and exo genes of the* λ *red bacteriophage*	Datsenko & Wanner 2000

Table S3: Primers used in this study.

Name	Sequence 5'-3' (in **bold** the portion annealing to the template, pSUB11)	Source
p354-clpX-FLAG-fwd	GCTGATTTACGGCAAACCGGAAGCGCAGGCTTCTGGCGAA**GACTACAAAGACCATGACG**	This study
p355-clpX-FLAG-rev	ATCCCCCCTTTTTTGGCTAACTGATTGTATGAATGTTTAA**CATATGAATATCCTCCTTAG**	This study
p181-cysN-FLAG-fwd	GGACGCCCGAGATTTGCTGGGAGATAAACATGGCGCTGCA **GACTACAAAGACCATGACG**	This study
p182-cysN-FLAG-rev	GGCGACAGTAACGGGATGAGAGTGCCAGACCACGTTCTCATCATGCAGCGCCATG**CATATGAATATCCTCCTTAG**	This study
p350-dnaJ-FLAG-fwd	CTTTGACGGCGTGAAAAAATTCTTTGACGATTTGACTCG**GACTACAAAGACCATGACG**	This study
p351-dnaJ-FLAG-rev	GATATACACCCGGGCTGAAGAAAAATACAACGGGAAAAGA**CATATGAATATCCTCCTTAG**	This study
p358-ubiG-FLAG-fwd	AGACGTTAACTACATGTTGCATACCCGCGCTAAAAAAGCC**GACTACAAAGACCATGACG**	This study
p359-ubiG-FLAG-rev	CGATGATCTAACGCAACCCTTATAGGAAAATTCTTTGATG**CATATGAATATCCTCCTTAG**	This study
p157-YigA-FLAG-fwd	GATGCTGCCGGAACTGCTGGAACGCTGGATTAAACGCGTA**GACTACAAAGACCATGACG**	This study
p158-YigA-FLAG-rev	CGCAGGAACCGCGAGACATCCTGAGAAAGCGCGACATCCGTCA**CATATGAATATCCTCCTTAG**	This study

2 Results

2.1 Parameter Tuning of TriPepSVM

For each tested combination of the class weights $W+$ and $W-$ we test a range of values for k and cost C in the cross-validation loop, and select at the end the combination of hyper-parameters which maximizes the model's performance, computed as balanced accuracy (see Supplementary Paragraph 1.3.3). We detect for both Human and *Salmonella* the optimal class weights of $W+ = 1.8$ and $W- = 0.2$, whereas *E.coli* performs best using $W+ = 1$ and $W- = 0.1$. However, it

has to be noticed that often more than one combination of parameters perform well in the cross-validation procedure, yielding little difference (about 1%) in the balanced accuracy compared to the best-performing parameter combinations.

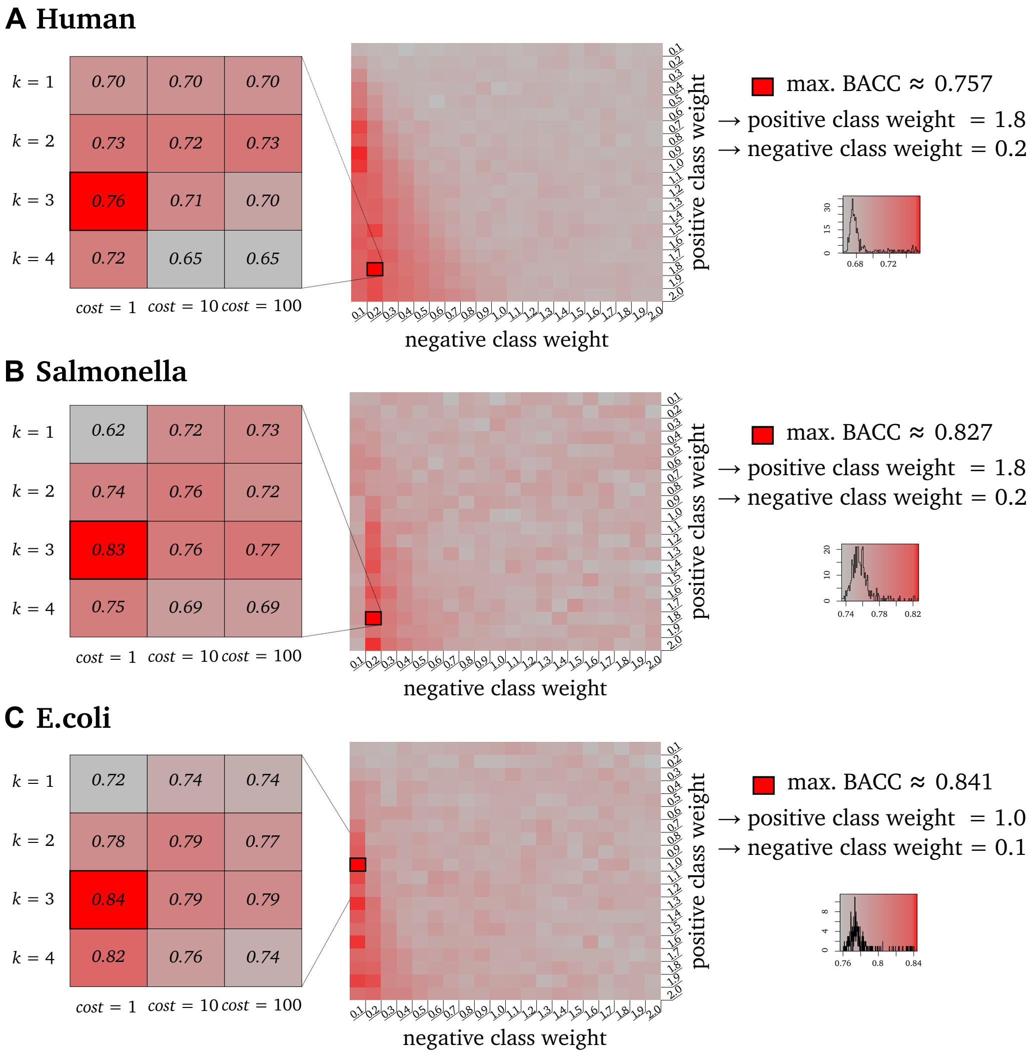

Figure S3: **Parameter tuning results.** The results from the parameter tuning procedure are shown for **(A)** human, **(B)** Salmonella and **(C)** E.coli. The heatmaps on the right hand side show the best average balanced accuracy value for all class weight combinations of $W+ = W- = 0.1, ..., 2$. Each cell represents a parameter tuning experiment with fixed class weights but different combinations of k and *cost*. The heatmaps on the left hand side report the average balanced accuracy for different combinations of $k = 1, 2, 3, 4$ and $cost = 1, 10, 100$, at the optimal fixed values of $W+$ and $W-$. The 10-fold cross-validation reports $k = 3$ and $cost = 1$ as optimal hyper parameters for human, *Salmonella* and *E.coli*.

2.2 Performance Comparison between different method

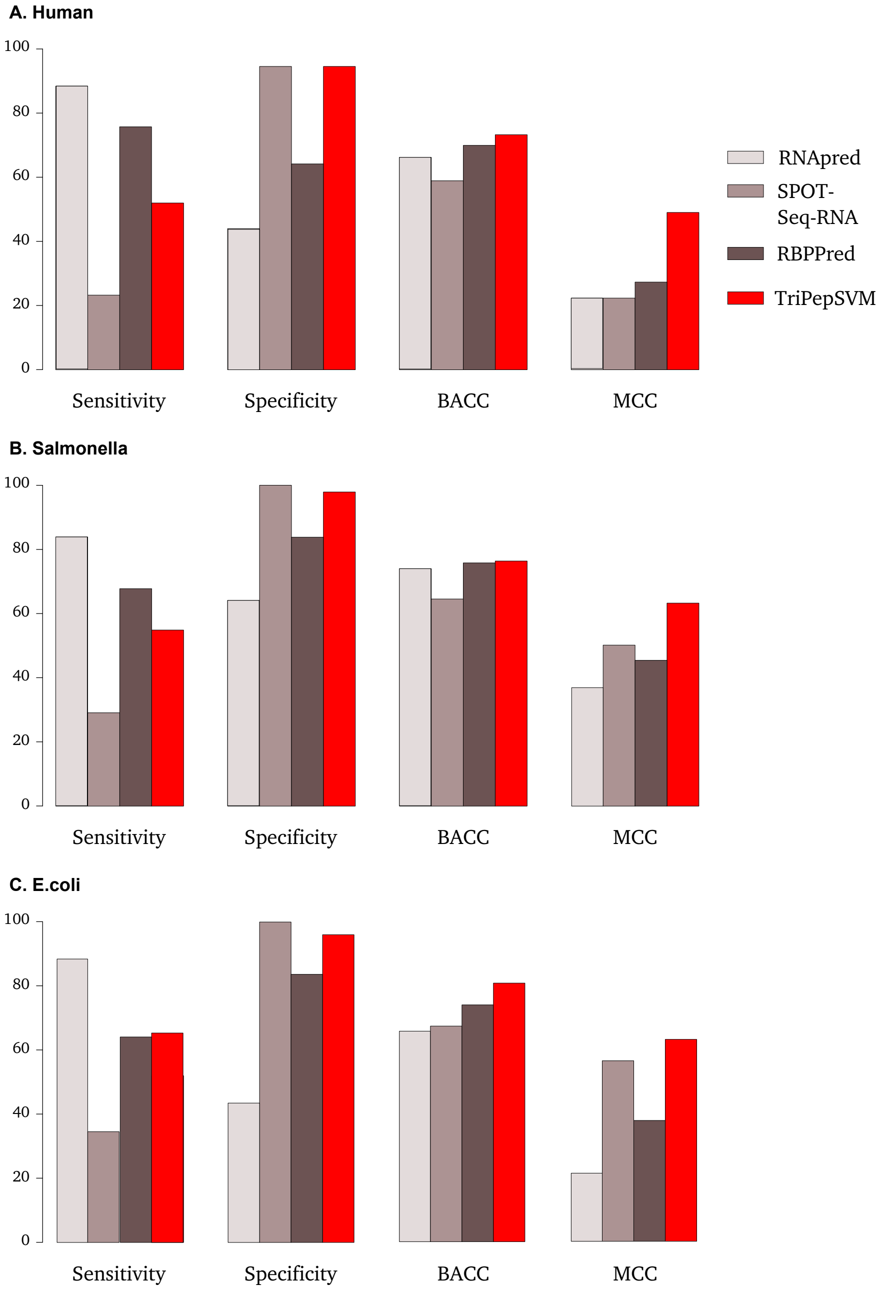

Figure S4: **Performance comparison between different methods.** The figure illustrates the performance comparisons between previously developed computational prediction methods (RNApred, SPOT-Seq-RNA, RBPPred) and our tool TriPepSVM. The performance measurements are computed on the test data set using the optimal thresholds for each taxon and tool, determined as described in subsection 1.4 of this file. The figure depicts the achieved sensitivity, specificity, balanced accuracy and MCC for all applications. TripepSVM performs with the best balanced accuracy and MCC in **(A)** human **(B)** *Salmonella* and **(C)** *E.coli*

2.3 Dealing with class imbalance

The training data for TriPepSVM is heavily imbalanced (see table 1, main text). Different strategies have been proposed to deal with this problem, the most popular being under-sampling of the majority class or adapting the importance of samples from the minority class (class-weighted SVMs). While we decided for the latter approach to account for class imbalance in our data, we also wanted to assess how well an under-sampling approach would perform in this situation.

Therefore, we randomly sampled n proteins from the positive and the negative data sets, where n corresponds to the number of training examples from the minority class, the positive class. This way, the performance of our model depends on the choice of the negative training set and is no longer deterministic. We therefore used a *bootstrapping* approach in which we repeated the sub-sampling, training and prediction on the test set for 10 times and averaged the performance over ten runs.

Figure S5 shows the performance on our test set in a similar fashion to main Figure 2. The performance of TriPepSVM does not change significantly when employing a different strategy to deal with with class imbalance. The slightly lower performance of the balanced training compared to the class-weighted SVM is most likely due to the reduced size of the negative set for each run of the bootstrapping procedure.

Furthermore, the bootstrapping approach allows us to assess the variance of training with different negative sets. Interestingly, the variance is small for Human and *E.Coli* but higher for *Salmonella*, as expected given the much smaller size of the training data set, compared to the other two organisms.

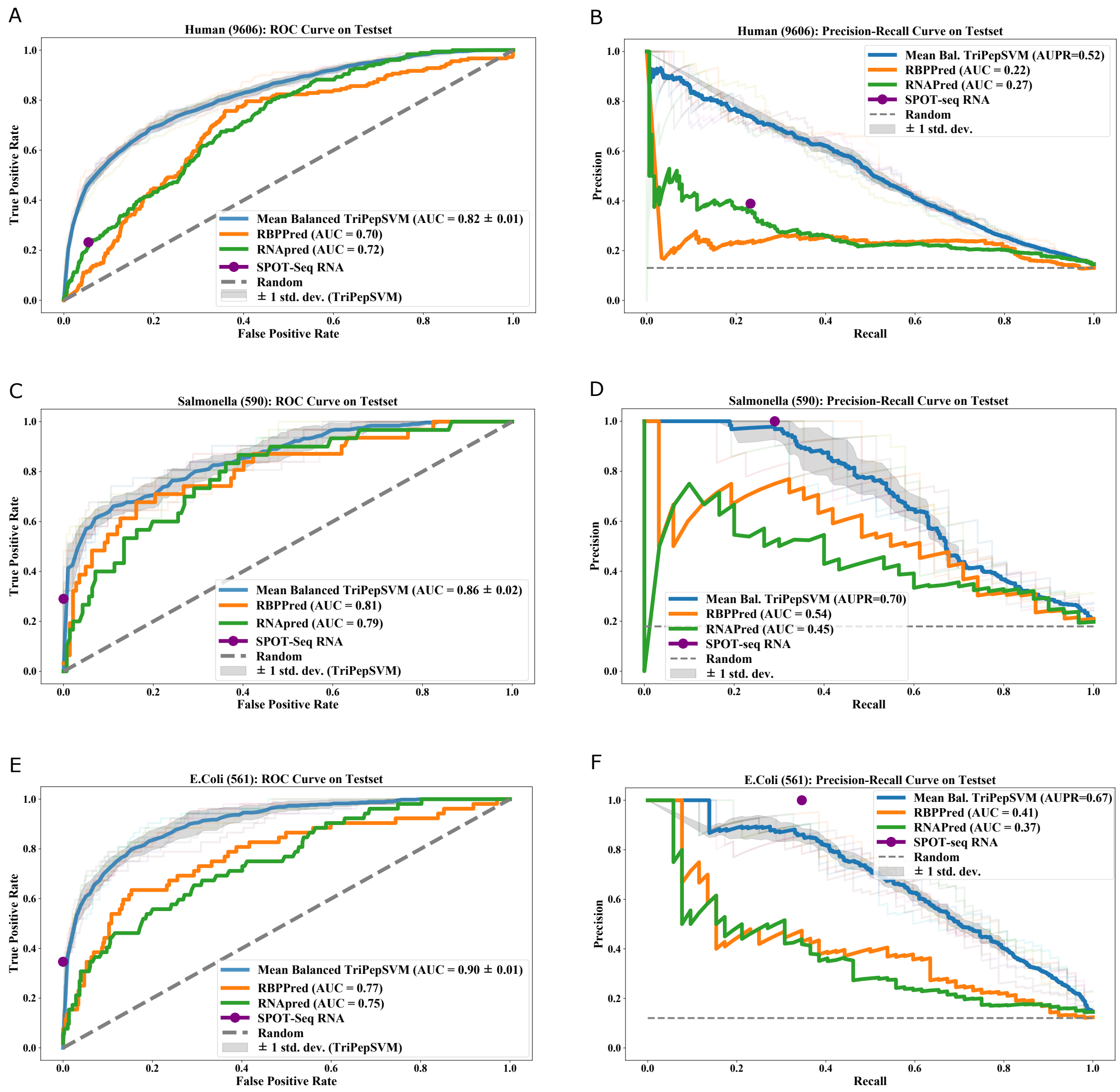

Figure S5: **Performance of TriPepSVM using a balanced training set.** Evaluation of the performance of TriPepSVM in comparison to other methods when trained on a balanced, subsampled data set. Reported are the mean ROC and PR curves from 10 consecutive runs. The grey area denotes one standard deviation. Each row in the figure corresponds to an organism (panel **A** & **B** for Human, **C** & **D** for *Salmonella* and **E** & **F** for *E.Coli*). SPOT-seq-RNA only outputs a class and no probability or score associated with the predictions and is hence represented only as a dot in the PR/ROC curves.

2.4 Runtime analysis

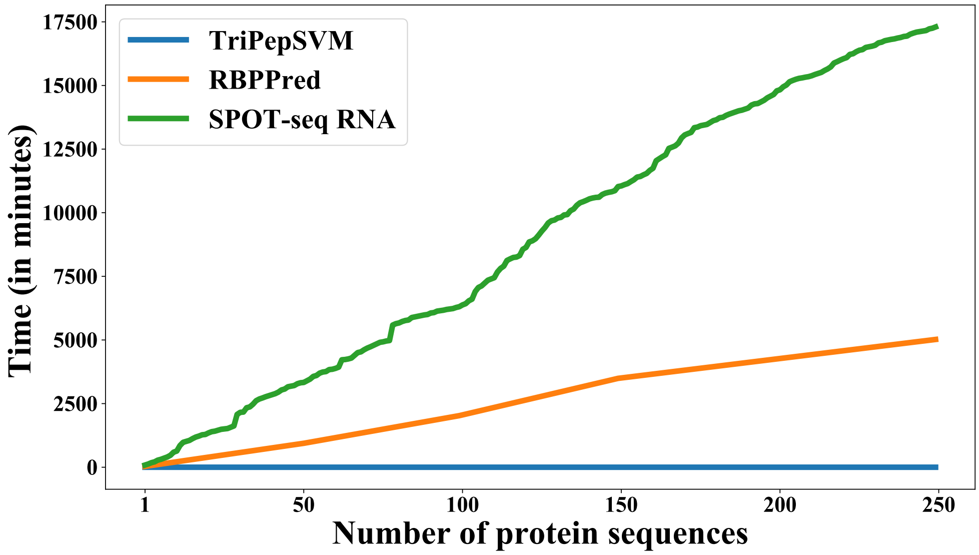

Figure S6: **Runtime comparison.** We assessed the runtime of TriPepSVM (blue, pre-trained), RBPpred (orange) and SPOT-seq-RNA (green) as a function of the number of predicted protein sequences on human (up to 250 proteins). We excluded RNAPred from this analysis because the application is not available for command line application. However, the RNApred web-server usually reports predictions within seconds. In our experiment TriPepSVM performed significantly faster compared to SPOT-seq-RNA and RBPPred, with a constant runtime (around 5 s) despite the increasing number of predicted proteins. In contrast, SPOT-seq-RNA and RBPPred require around 288 and 83 hours, respectively, for the prediction of 250 proteins. All reported times were collected on a single core (2.2 GHz Supermicro 2023US-TR4, 1 TB RAM).

2.5 Performance Comparison: Cross-species prediction

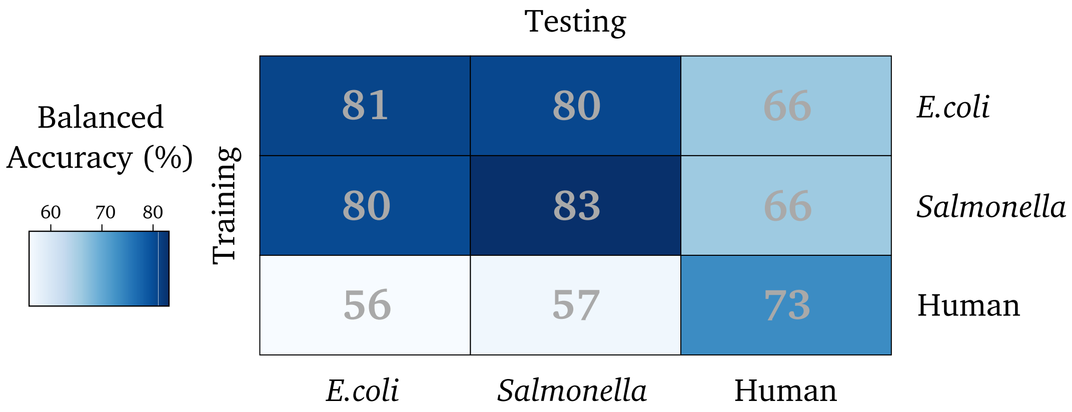

Figure S7: **Cross-species performance evaluation of TriPepSVM.** Shown is the performance of TriPepSVM, measured as balanced accuracy (as defined in paragraph 1.3.3), when trained separately on each organism (*E.coli*, *Salmonella* and human) and evaluated on the test set of either the same or another organism (cross-species). Rows are the organisms which the classifier was trained on, and the columns are the organisms which the classifier was tsted on. When training on an organism and testing on another we exclude from the test set sequences with more than 90% sequence similarity to the sequences of the training set. This way, we collect 40 RBPs and 327 non-RBPs for *E.coli*, 19 RBPs and 92 non-RBPs for *Salmonella* and 180 RBPs and 1,204 non-RBPs for human.

2.6 Overlap between cross-species predictions

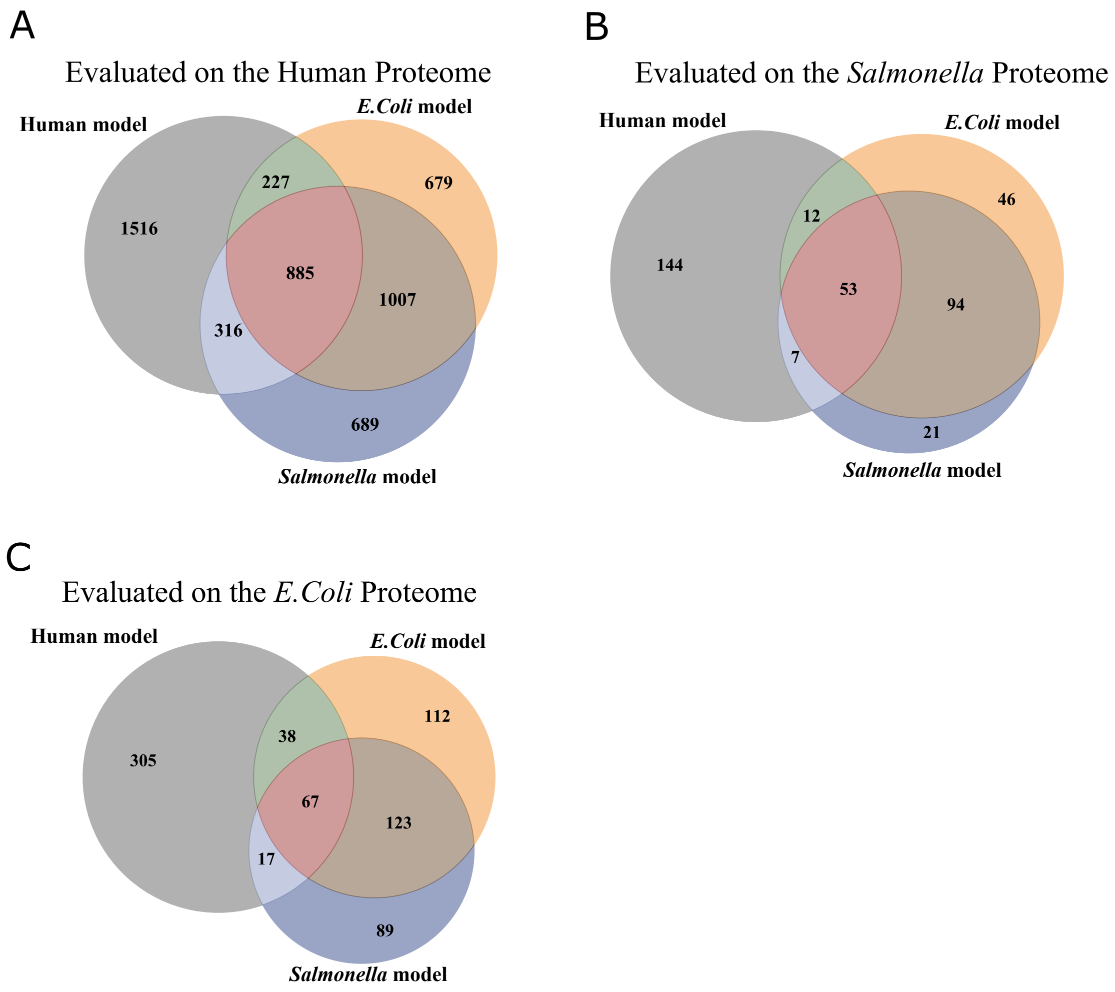

Figure S8: **Cross-species prediction.** We trained three TriPepSVM models on human, *Salmonella* and *E.Coli* and performed proteome-wide predictions with all three models on A) the human proteome, B) the *Salmonella* proteome and C) the *E.Coli* proteome. Shown is the overlap of the three classifiers, in terms of predicted RBPs, for each organism separately.

Table S4: **Uniprot keywords used to remove nucleotide-binding associated proteins.**

Uniprot Keyword	Definition	ID
Activator	Protein that positively regulates either the transcription of one or more genes, or the translation of mRNA.	KW-0010
ADP-ribosylation	Protein which is post-translationally modified by the attachment of at least one ADP-ribosyl group.	KW-0013
Chromatin regulator	Protein controlling the opening or closing of chromatin.	KW-0156
Chromosome partition	Protein involved in chromosome partition, the process by which newly replicated plasmids and chromosomes are actively segregated prior to cell division.	KW-0159
Nucleosome core	Protein characteristic of the nucleosome, a repeating structural unit in chromatin that packages DNA to give the chromatin a 'beads-on-a-string' appearance.	KW-0544
Chromosome	Protein which is associated with chromosomal DNA, including histones, protamines and high mobility group proteins.	KW-0158
Endonuclease	Phosphodiesterase capable of cleaving at phosphodiester internal bonds within a DNA or RNA substrate.	KW-0255
Excision nuclease	Enzyme which excises abnormal or mismatched nucleotides from a DNA strand.	KW-0267
Exonuclease	Enzyme that degrades DNA or RNA by progressively splitting off single nucleotides from one end of the chain.	KW-0269
Helicase	Protein with an helicase activity. Helicases are ATPases that catalyze the unwinding of double-stranded nucleic acids. They are tightly integrated (or coupled) components of various macromolecular complexes which are involved in processes such as DNA replication, recombination, and nucleotide excision repair, as well as RNA transcription and splicing.	KW-0347
Intron homing	Endonucleases involved in intron homing, a genetic event leading to the transfer of an intron DNA sequence. This type of intron mobility depends on site-specific restriction endonucleases encoded by the mobile introns.	KW-0404
Isomerase	Enzyme that catalyzes the 1,1-, 1,2- or 1,3-hydrogen shift. The 1,1-hydrogen shift is an inversion at an asymmetric carbon center (racemases, epimerases). The 1,2-hydrogen shift involved a hydrogen transfer between two adjacent carbon atoms, one undergoing oxidation, the other reduction (aldose-ketose isomerases). The 1,3-hydrogen shifts are allylic or azaallylic (when nitrogen is one of the three atoms) isomerizations.	KW-0413
Nuclease	Enzyme that degrades nucleic acids into shorter oligonucleotides or single nucleotide subunits by hydrolyzing sugar-phosphate bonds in the nucleic acid backbone.	KW-0540
Spliceosome	Protein of the spliceosome, a very large complex of small nuclear RNA/protein particles (snRNPs) which assemble with pre-mRNA to achieve RNA splicing.	KW-0747
Topoisomerase	Enzymes capable of altering the degree of supercoiling of double-stranded DNA molecules. Various topoisomerases can increase or relax supercoiling, convert single-stranded rings to intertwined double-stranded rings, tie and untie knots in single stranded and duplex rings or catenate and decatenate duplex rings. Any enzyme that cleaves only one strand of a DNA duplex and then reseals it is classified as a type I topoisomerase (Topo I). Type II topoisomerases (Topo II) change DNA topology by breaking and rejoining double-stranded DNA.	KW-0799
Transcription	Protein involved in the transfer of genetic information from DNA to messenger RNA (mRNA) by DNA-directed RNA polymerase. In the case of some RNA viruses, protein involved in the transfer of genetic information from RNA to messenger RNA (mRNA) by RNA-directed RNA polymerase.	KW-0804
Transcription regulation	Protein involved in the regulation of the transcription process.	KW-0805
Transcription termination	Protein involved in transcription termination.	KW-0806
Translation regulation	Protein involved in the regulation of the transcription process. Category	KW-0810
DNA-binding	Protein which binds to DNA, typically to pack or modify the DNA, or to regulate gene expression. Among those proteins that recognize specific DNA sequences, there are a number of characteristic conserved motifs believed to be essential for specificity. Many DNA-binding domains are described in PROSITE.	KW-0238

DNA damage	Protein induced by DNA damage or protein involved in the response to DNA damage. Drug- or radiation-induced injuries in DNA introduce deviations from its normal double-helical conformation. These changes include structural distortions which interfere with replication and transcription, as well as point mutations which disrupt base pairs and exert damaging effects on future generations through changes in DNA sequence. Response to DNA damage results in either repair or tolerance.	KW-0227
DNA excision	Protein involved in the repair of damages to one strand of DNA (loss of purines due to thermal fluctuations, formation of pyrimidine dimers by UV irradiation, for instance). The site of damage is recognized, excised by an endonuclease, the correct sequence is copied from the complementary strand by a polymerase and the ends of this correct sequence are joined to the rest of the strand by a ligase. In bacterial systems, the polymerase also acts as endonuclease. Excisase A and other proteins involved in recombination mediate DNA excision; a process whereby abnormal or mismatched nucleotides are enzymatically cut out of a strand of a DNA molecule.	KW-0228
DNA integration	Protein involved in DNA integration, a process that mediates the insertion of foreign genetic material, or other duplex DNA, into a chromosome, or another replicon, in order to form a covalently linked DNA continuous with the host DNA.	KW-0229
DNA repair	Protein involved in the repair of DNA, the various biochemical processes by which damaged DNA can be restored. DNA repair embraces, for instance, not only the direct reversal of some types of damage (such as the enzymatic photoreactivation of thymine dimers), but also multiple distinct mechanisms for excising damaged base; termed nucleotide excision repair (NER), base excision repair (BER) and mismatch repair (MMR); or mechanisms for repairing double-strand breaks.	KW-0234
DNA replication	Protein involved in DNA replication, i.e. the duplication of DNA by making a new copy of an existing molecule. The parental double-stranded DNA molecule is replicated semi conservatively, i.e. each copy contains one of the original strands paired with a newly synthesized strand that is complementary in terms of AT and GC base pairing.	KW-0235
DNA replication inhibitor	Protein involved in the inhibition of DNA replication.	KW-0236
DNA synthesis	Protein involved in the synthesis of DNA from deoxyribonucleic acid monomers.	KW-0237
DNA recombination	Protein involved in DNA recombination, i.e. any process in which DNA molecules are cleaved and the fragments are rejoined to give a new combination.	KW-0233
DNA-directed DNA polymerase	Enzyme that catalyzes DNA synthesis by addition of deoxyribonucleotide units to a DNA chain using DNA as a template. They can also possess exonuclease activity and therefore function in DNA repair.	KW-0239
DNA-directed RNA polymerase	Protein of the DNA-directed RNA polymerase complexes, which catalyze RNA synthesis the by addition of ribonucleotide units to a RNA chain using DNA as a template. They can initiate a chain de novo. Prokaryotes have a single enzyme for the three RNA types that is subject to stringent regulatory mechanisms. Eukaryotes have type I that synthesizes all rRNA except the 5S component, type II that synthesizes mRNA and hnRNA and type III that synthesizes tRNA and the 5S component of rRNA.	KW-0240
RNA-binding	Protein which binds to RNA.	KW-0694
RNA-directed DNA polymerase	Enzyme (EC 2.7.7.49) which synthesizes (-)DNA on a (+)RNA template. They are encoded by the pol gene of retroviruses and by certain retrovirus-like elements.	KW-0695
RNA-directed RNA polymerase	Enzyme (EC 2.7.7.48) which synthesizes (+)RNA on a (-)RNA template. They are encoded by many viruses.	KW-0696
RNA repair	Protein involved in the repair of RNA, the various biochemical processes by which damaged RNA can be restored.	KW-0692
rRNA-binding	Protein which binds to ribosomal RNA.	KW-0699
rRNA processing	Protein involved in the processing of the primary rRNA transcript to yield a functional rRNA. This includes the cleavage and other modifications.	KW-0698
mRNA processing	Protein involved in the processing of the primary mRNA transcript to yield a functional mRNA. This includes 5' capping, 3' cleavage and polyadenylation, as well as mRNA splicing and RNA editing.	KW-0507

mRNA splicing	Protein involved in the process by which nonsense sequences or intervening sequences (introns) are removed from pre-mRNA to generate a functional mRNA (messenger RNA) that contains only exons.	KW-0508
mRNA transport	Protein which is involved in the mechanism of export of mRNAs from the nucleus to the cytoplasm.	KW-0509
tRNA-binding	Protein which binds transfer RNA, for example some ribosomal proteins or some aminoacyl-tRNA synthetases.	KW-0820
tRNA processing	Protein involved in the processing of the primary tRNA transcript to yield a functional tRNA. Transcription of tRNA genes results in a large precursor molecule which may even contain sequences for several tRNA molecules. This primary transcript is subsequently processed by cleavage and by modification of the appropriate bases.	KW-0819
Ribonucleoprotein	Proteins conjugated with ribonucleic acid (RNA). Ribonucleoprotein are involved in a wide range of cellular processes. Besides ribosomes, in eukaryotic cells both initial RNA transcripts in the nucleus (hnRNA) and cytoplasmic mRNAs exist as complexes with specific sets of proteins. Processing (splicing) of the former is carried out by small nuclear RNPs (snRNPs). Other examples are the signal recognition particle responsible for targetting proteins to endoplasmic reticulum and a complex involved in termination of transcription.	KW-0687
Ribosomal protein	Protein of the ribosome, large ribonucleoprotein particles where the translation of messenger RNA (mRNA) into protein occurs. They are both free in the cytoplasm and attached to membranes of eukaryotic and prokaryotic cells. Ribosomes are also present in all plastids and mitochondria, where they translate organelle-encoded mRNA.	KW-0689
Viral genome packagingn	Protein involved in actively packaging the replicated viral genome into a protective shell or envelope. Such packaging proteins are present for example in adenoviruses, herpesviruses and tailed bacteriophages. In bacteriophages, the packaging proteins complex is involved in recognizing and selecting a neo-synthesized viral genome in order to translocate it into a pre-assembled empty procapsid. DNA cleavage is sometimes coupled to genome packaging as well as maturation steps that induce structural changes in the assembled capsid.	KW-0231
Viral RNA replication	Viral protein involved in the SYnthesis of multiple copies of the viral RNA genome. The replicated genomes provide support for further viral transcription or are assembled into progeny virions.	KW-0693
Ribosome biogenesis	Protein involved in the synthesis of ribosomes.	KW-0690
Nucleotide-binding	Protein which binds a nucleotide, a phosphate ester of a nucleoside consisting of a purine or pyrimidine base linked to ribose or deoxyribose phosphates.	KW-0547

Table S5: **QuickGO terms used to remove nucleotide-binding associated proteins.**

QuickGO term	Definition	ID
RNA binding	Interacting selectively and non-covalently with an RNA molecule or a portion thereof.	GO:0003723
DNA binding	Any molecular function by which a gene product interacts selectively and non-covalently with DNA (deoxyribonucleic acid).	GO:0003677
nucleotide binding	Interacting selectively and non-covalently with a nucleotide, any compound consisting of a nucleoside that is esterified with (ortho)phosphate or an oligophosphate at any hydroxyl group on the ribose or deoxyribose.	GO:0000166

Table S6: **Pfam domains associated with RNA, DNA or nucleotide-binding.** The table contains 526 HMM models, e.g. domains, from the Pfam data base. We include models where (1) the description matches the pattern "RNA binding/recognition or processing", (2) the PDB identifier of the PDB-Pfam mapping is annotated as RBD, (3) annotated RBDs from QuickGO and (4) classical RBDs from literature. We apply the domain-based prediction in two different modes. In the data collection pipeline we include all models to ensure the removal of all potential RBPs. However, the set contains a lot of false positive matches as we include putative RBDs. In the second mode we apply a more conservative set of models (219 bold marked Pfam domains) for predicting RBPs based on Pfam domains, excluding putative RBDs.

Pfam ID	Pfam name	Pfam ID	Pfam name	Pfam ID	Pfam name
**PF00009**	GTP_EFTU	**PF00013**	KH_1	PF00023	Ank
**PF00035**	dsrm	PF00047	ig	PF00069	Pkinase
PF00075	RNase_H	**PF00076**	RRM_1	PF00078	RVT_1
**PF00096**	zf-C2H2	PF00098	zf-CCHC	**PF00133**	tRNA-synt_1
PF00134	Cyclin_N	PF00136	DNA_pol_B	PF00140	Sigma70_r1_2
PF00152	tRNA-synt_2	**PF00163**	Ribosomal_S4	PF00164	Ribosom_S12_S23
PF00169	PH	PF00177	Ribosomal_S7	**PF00181**	Ribosomal_L2
PF00189	Ribosomal_S3_C	PF00203	Ribosomal_S19	PF00237	Ribosomal_L22
PF00238	Ribosomal_L14	PF00252	Ribosomal_L16	PF00253	Ribosomal_S14
**PF00270**	DEAD	PF00271	Helicase_C	PF00276	Ribosomal_L23
PF00281	Ribosomal_L5	PF00297	Ribosomal_L3	**PF00298**	Ribosomal_L11
PF00312	Ribosomal_S15	**PF00313**	CSD	PF00318	Ribosomal_S2
PF00327	Ribosomal_L30	**PF00333**	Ribosomal_S5	PF00338	Ribosomal_S10
**PF00347**	Ribosomal_L6	PF00366	Ribosomal_S17	PF00380	Ribosomal_S9
PF00398	RrnaAD	PF00400	WD40	PF00410	Ribosomal_S8
PF00411	Ribosomal_S11	**PF00416**	Ribosomal_S13	PF00444	Ribosomal_L36
**PF00445**	Ribonuclease_T2	PF00448	SRP54	**PF00453**	Ribosomal_L20
PF00466	Ribosomal_L10	PF00467	KOW	PF00468	Ribosomal_L34
PF00471	Ribosomal_L33	PF00472	RF-1	**PF00536**	SAM_1
PF00542	Ribosomal_L12	**PF00545**	Ribonuclease	PF00562	RNA_pol_Rpb2_6
PF00563	EAL	PF00572	Ribosomal_L13	PF00573	Ribosomal_L4
**PF00575**	S1	PF00587	tRNA-synt_2b	**PF00588**	SpoU_methylase
**PF00598**	Flu_M1	**PF00600**	Flu_NS1	**PF00603**	Flu_PA
**PF00604**	Flu_PB2	PF00615	RGS	PF00623	RNA_pol_Rpb1_2
PF00631	G-gamma	**PF00636**	Ribonuclease_3	PF00641	zf-RanBP
PF00642	zf-CCCH	**PF00658**	PABP	PF00673	Ribosomal_L5_C
PF00679	EFG_C	**PF00680**	RdRP_1	PF00687	Ribosomal_L1
PF00707	IF3_C	**PF00749**	tRNA-synt_1c	PF00753	Lactamase_B
**PF00806**	PUF	**PF00825**	Ribonuclease_P	PF00827	Ribosomal_L15e
PF00828	Ribosomal_L18e	PF00829	Ribosomal_L21p	PF00830	Ribosomal_L28
PF00831	Ribosomal_L29	PF00832	Ribosomal_L39	PF00833	Ribosomal_S17e
PF00843	Arena_nucleocap	**PF00849**	PseudoU_synth_2	PF00861	Ribosomal_L18p
**PF00874**	PRD	PF00886	Ribosomal_S16	PF00900	Ribosomal_S4e
**PF00910**	RNA_helicase	PF00922	Phosphoprotein	PF00929	RNase_T
PF00935	Ribosomal_L44	PF00945	Rhabdo_ncap	**PF00949**	Peptidase_S7
**PF00978**	RdRP_2	**PF00981**	Rota_NS53	**PF00998**	RdRP_3
PF01000	RNA_pol_A_bac	**PF01005**	Flavi_NS2A	PF01015	Ribosomal_S3Ae
PF01016	Ribosomal_L27	PF01020	Ribosomal_L40e	**PF01021**	TYA
**PF01029**	NusB	PF01084	Ribosomal_S18	PF01090	Ribosomal_S19e
PF01092	Ribosomal_S6e	PF01096	TFIIS_C	**PF01132**	EFP
PF01135	PCMT	PF01138	RNase_PH	**PF01142**	TruD
PF01157	Ribosomal_L21e	PF01158	Ribosomal_L36e	PF01159	Ribosomal_L6e
PF01161	PBP	PF01165	Ribosomal_S21	**PF01176**	eIF-1a
PF01191	RNA_pol_Rpb5_C	PF01192	RNA_pol_Rpb6	PF01193	RNA_pol_L
PF01194	RNA_pol_N	PF01196	Ribosomal_L17	PF01197	Ribosomal_L31
PF01198	Ribosomal_L31e	PF01199	Ribosomal_L34e	PF01200	Ribosomal_S28e
PF01201	Ribosomal_S8e	PF01202	SKI	PF01245	Ribosomal_L19
PF01246	Ribosomal_L24e	PF01247	Ribosomal_L35Ae	PF01248	Ribosomal_L7Ae
PF01249	Ribosomal_S21e	**PF01250**	Ribosomal_S6	PF01251	Ribosomal_S7e
PF01253	SUI1	**PF01269**	Fibrillarin	PF01272	GreA_GreB
PF01280	Ribosomal_L19e	PF01281	Ribosomal_L9_N	PF01282	Ribosomal_S24e
PF01283	Ribosomal_S26e	**PF01287**	eIF-5a	PF01294	Ribosomal_L13e
**PF01300**	Sua5_yciO_yrdC	**PF01336**	tRNA_anti-codon	PF01351.14	RNase_HII
PF01378	IgG_binding_B	PF01386	Ribosomal_L25p	PF01399	PCI
**PF01409**	tRNA-synt_2d	**PF01416**	PseudoU_synth_1	**PF01423**	LSM
**PF01472**	PUA	**PF01479**	S4	**PF01480**	PWI
PF01509	TruB_N	**PF01517**	HDV_ag	**PF01518**	PolyG_pol
**PF01588**	tRNA_bind	PF01599	Ribosomal_S27	PF01632	Ribosomal_L35p

**PF01649**	Ribosomal_S20p	**PF01652**	IF4E	PF01655	Ribosomal_L32e
PF01656	CbiA	**PF01660**	Vmethyltransf	PF01661	Macro
**PF01665**	Rota_NSP3	PF01667	Ribosomal_S27e	**PF01668**	SmpB
PF01693	Cauli_VI	PF01728	FtsJ	**PF01743**	PolyA_pol
**PF01746**	tRNA_m1G_MT	PF01765	RRF	PF01775	Ribosomal_L18ae
PF01776	Ribosomal_L22e	PF01777	Ribosomal_L27e	PF01779	Ribosomal_L29e
PF01780	Ribosomal_L37ae	PF01781	Ribosomal_L38e	PF01783	Ribosomal_L32p
**PF01787**	Ilar_coat	PF01796	OB_aCoA_assoc	**PF01798**	Nop
**PF01805**	Surp	**PF01806**	Paramyxo_P	**PF01818**	Translat_reg
PF01829	Peptidase_A6	**PF01868**	UPF0086	**PF01877**	RNA_binding
PF01878	EVE	PF01907	Ribosomal_L37e	PF01909	NTP_transf_2
PF01918	Alba	**PF01922**	SRP19	PF01926	MMR_HSR1
PF01929	Ribosomal_L14e	PF01938	TRAM	PF01978	TrmB
**PF01985**	CRS1_YhbY	**PF02005**	TRM	PF02037	SAP
**PF02081**	TrpBP	PF02097	Filo_VP35	**PF02123**	RdRP_4
**PF02137**	A_deamin	PF02150	RNA_POL_M_15KD	**PF02170**	PAZ
**PF02171**	Piwi	PF02198	SAM_PNT	**PF02290**	SRP14
**PF02295**	z-alpha	PF02492	cobW	**PF02509**	Rota_NS35
PF02568	ThiI	**PF02599**	CsrA	PF02609	Exonuc_VII_S
PF02792	Mago_nashi	**PF02854**	MIF4G	PF02881	SRP54_N
PF02912	Phe_tRNA-synt_N	**PF02926**	THUMP	**PF02978**	SRP_SPB
PF03104	DNA_pol_B_exo1	**PF03123**	CAT_RBD	PF03129	HGTP_anticodon
PF03143	GTP_EFTU_D3	PF03144	GTP_EFTU_D2	**PF03147**	FDX-ACB
PF03193	DUF258	PF03205	MobB	**PF03246**	Pneumo_ncap
PF03297	Ribosomal_S25	**PF03368**	Dicer_dimer		
PF03462	PCRF	PF03463	eRF1_1	PF03464	eRF1_2
PF03465	eRF1_3	PF03467	Smg4_UPF3	PF03468	XS
**PF03483**	B3_4	**PF03484**	B5	PF03501	S10_plectin
PF03566	Peptidase_A21	PF03604	DNA_RNApol_7kD	PF03719	Ribosomal_S5_C
PF03725	RNase_PH_C	**PF03726**	PNPase	PF03764	EFG_IV
PF03828	PAP_assoc	**PF03854**	zf-P11	**PF03861**	ANTAR
PF03870	RNA_pol_Rpb8	PF03871	RNA_pol_Rpb5_N	PF03874	RNA_pol_Rpb4
PF03876	SHS2_Rpb7-N	**PF03880**	DbpA	PF03919	mRNA_cap_C
PF03939	Ribosomal_L23eN	PF03946	Ribosomal_L11_N	PF03947	Ribosomal_L2_C
PF03948	Ribosomal_L9_C	**PF03950**	tRNA-synt_1c_C	PF03979	Sigma70_r1_1
**PF04059**	RRM_2	**PF04135**	Nop10p	PF04146	YTH
PF04266	ASCH	PF04280	Tim44	PF04378	RsmJ
**PF04410**	Gar1	PF04452	Methyltrans_RNA	**PF04514**	BTV_NS2
**PF04522**	DUF585	PF04539	Sigma70_r3	PF04542	Sigma70_r2
PF04546	Sigma70_ner	PF04548	AIG1	**PF04557**	tRNA_synt_1c_R2
**PF04558**	tRNA_synt_1c_R1	PF04560	RNA_pol_Rpb2_7	PF04561	RNA_pol_Rpb2_2
PF04563	RNA_pol_Rpb2_1	PF04565	RNA_pol_Rpb2_3	PF04566	RNA_pol_Rpb2_4
PF04567	RNA_pol_Rpb2_5	PF04758	Ribosomal_S30	**PF04774**	HABP4_PAI-RBP1
**PF04818**	CTD_bind	**PF04845**	PurA	**PF04847**	Calcipressin
PF04851	ResIII	**PF04857**	CAF1	**PF04926**	PAP_RNA-bind
PF04983	RNA_pol_Rpb1_3	PF04990	RNA_pol_Rpb1_7	PF04992	RNA_pol_Rpb1_6
PF04997	RNA_pol_Rpb1_1	PF04998	RNA_pol_Rpb1_5	PF05000	RNA_pol_Rpb1_4
**PF0502**	DCP2	PF05046	Img2	PF05047	L51_S25_CI-B8
**PF05087**	Rota_VP2	PF05162	Ribosomal_L41	**PF05172**	Nup35_RRM
**PF05383**	La	**PF05413**	Peptidase_C34	PF05470	eIF-3c_N
PF05486	SRP9-21	**PF05634**	APO_RNA-bind	PF05697	Trigger_N
**PF05731**	TROVE	**PF05733**	Tenui_N	**PF05741**	zf-nanos
**PF05746**	DALR_1	**PF05788**	Orbi_VP1	**PF05890**	Ebp2
**PF06003**	SMN	PF06220	zf-U1	PF06293	Kdo
PF06414	Zeta_toxin	PF06467	zf-FCS	**PF06478**	Corona_RPol_N
PF06479	Ribonuc_2-5A	PF06747	CHCH	PF06815	RVT_connect
PF06817	RVT_thumb	PF06984	MRP-L47	**PF06991**	MFAP1
PF07147	PDCD9	PF07296	TraP	PF07447	VP40
**PF07497**	Rho_RNA_bind	PF07498	Rho_N	PF07500	TFIIS_M
PF07521	RMMBL	**PF07541**	EIF_2_alpha	PF07647	SAM_2
**PF07650**	KH_2	PF07654	C1-set	PF07679	I-set
PF07686	V-set	PF07714	Pkinase_Tyr	**PF07717**	OB_NTP_bind
**PF07925**	RdRP_5	**PF08032**	SpoU_sub_bind	PF08069	Ribosomal_S13_N
PF08071	RS4NT	PF08079	Ribosomal_L30_N	PF08080	zf-RNPHF
**PF08144**	CPL	**PF08147**	DBP10CT	**PF08152**	GUCT
**PF08167**	RIX1	**PF08190**	PIH1	PF08205	C2-set_2
PF08206	OB_RNB	PF08213	DUF1713	PF08228	RNase_P_pop3
**PF08264**	Anticodon_1	**PF08289**	Flu_M1_C	PF08292	RNA_pol_Rbc25
PF08293	MRP-S33	PF08433	KTI12	**PF08492**	SRP72
**PF08517**	AXH	**PF08524**	rRNA_processing	PF08561	Ribosomal_L37
**PF08572**	PRP3	PF08662	eIF2A	**PF08675**	RNA_bind

**PF08698**	Fcf2	PF08699	ArgoL1	**PF08710**	nsp9
**PF08777**	RRM_3	PF08798	CRISPR_assoc	**PF08799**	PRP4
**PF08845**	SymE_toxin	**PF09000**	Cytotoxic	PF09105	SelB-wing_1
**PF09106**	SelB-wing_2	**PF09107**	SelB-wing_3	**PF09142**	TruB_C
**PF09157**	TruB-C_2	**PF09162**	Tap-RNA_bind	PF09173	eIF2_C
**PF09190**	DALR_2	PF09235	Ste50p-SAM	**PF09246**	PHAT
**PF09334**	tRNA-synt_1g	**PF09387**	MRP	**PF09401**	NSP10
PF09405	Btz	PF09598	Stm1_N	**PF09738**	DUF2051
PF09776	Mitoc_L55	PF09809	MRP-L27	PF09812	MRP-L28
**PF10133**	RNA_bind_2	PF10147	CR6_interact	**PF10150**	RNase_E_G
PF10210	MRP-S32	PF10213	MRP-S28	PF10236	DAP3
PF10244	MRP-L51	PF10245	MRP-S22	PF10246	MRP-S35
**PF10258**	RNA_GG_bind	**PF10273**	WGG	PF10283	zf-CCHH
**PF10288**	CTU2	**PF10373**	EST1_DNA_bind	PF10385	RNA_pol_Rpb2_45
**PF10447**	EXOSC1	**PF10458**	Val_tRNA-synt_C	**PF10477**	EIF4E-T
PF10484	MRP-S23	PF10501	Ribosomal_L50	**PF10567**	Nab6_mRNP_bdg
**PF10597**	U5_2-snRNA_bdg	**PF10598**	RRM_4	PF10780	MRP_L53
**PF10789**	Phage_RpbA	PF10996	Beta-Casp	**PF11435**	She2p
PF11438	N36	**PF11473**	B2	PF11648	RIG-I_C-RD
**PF11717**	Tudor-knot	**PF11718**	CPSF73-100_C	PF11788	MRP-L46
**PF11955**	PORR	**PF11969**	DcpS_C	**PF12009**	Telomerase_RBD
**PF12171**	zf-C2H2_jaz	**PF12212**	PAZ_siRNAbind	PF12220	U1snRNP70_N
**PF12235**	FXMRP1_C_core	PF12328	Rpp20	**PF12627**	PolyA_pol_RNAbd
PF12701	LSM14	PF12706	Lactamase_B_2	**PF12745**	HGTP_anticodon2
PF12796	Ank_2	PF12862	ANAPC5	PF12869	tRNA_anti-like
PF12872	OST-HTH	**PF12923**	RRP7	PF12961	DUF3850
**PF13014**	KH_3	PF13017	Maelstrom	**PF13083**	KH_4
PF13086	AAA_11	PF13087	AAA_12	**PF13184**	KH_5
**PF13234**	rRNA_proc-arch	PF13238	AAA_18	PF13245	AAA_19
PF13395	HNH_4	**PF13397**	RbpA	PF13509	S1_2
PF13543	KSR1-SAM	PF13603	tRNA-synt_1_2	**PF13636**	Nol1_Nop2_Fmu_2
PF13637	Ank_4	PF13656	RNA_pol_L_2	PF13671	AAA_33
PF13680	DUF4152	**PF13725**	tRNA_bind_2	**PF13742**	tRNA_anti_2
PF13857	Ank_5	PF13869	NUDIX_2	**PF13893**	RRM_5
PF13895	Ig_2	PF13927	Ig_3	**PF13958**	ToxN_toxin
PF14204	Ribosomal_L18_c	**PF14259**	RRM_6	PF14306	PUA_2
PF14374	Ribos_L4_asso_C	PF14392	zf-CCHC_4	PF14438	SM-ATX
**PF14444**	S1-like	PF14492	EFG_II	PF14580	LRR_9
**PF14608**	zf-CCCH_2	**PF14622**	Ribonucleas_3_3	PF14693	Ribosomal_TL5_C
**PF14709**	DND1_DSRM	PF14943	MRP-S26	PF14955	MRP-S24
PF14978	MRP-63	**PF15247**	SLBP_RNA_bind	**PF15313**	HEXIM
**PF15320**	RAM	PF15433	MRP-S31	**PF15608**	PELOTA_1
**PF15777**	Anti-TRAP	**PF15801**	zf-C6H2	**PF15985**	KH_6
**PF16005**	MOEP19	**PF16367**	RRM_7	**PF16482**	Staufen_C
**PF16520**	BDV_M	**PF16651**	RRM_u2	**PF16780**	AIMP2_LysRS_bd
**PF16842**	RRM_occluded	**PF16852**	HHV-1_VABD	**PF16969**	SRP68
**PF14605**	Nup35_RRM_2				

2.7 Feature Selection

Table S7: **Important tripetides from the human SVM model.** The top 50 important tripeptides from the human SVM model, i.e. the ones that contributed the most to the classification of human proteins into RBPs versus non-RBPs are ranked according to their absolute value of the SVM weight, computed as described in the Method section.

Tripeptide	frequency in disordered regions of RBPs	SVM weight
LLL	0.075	-2.83
GKT	0.24	2.63
LLK	0.13	2.33

KNL	0.17	2.11
CGK	0.067	2.09
MAA	0.25	1.84
LLF	0.07	-1.79
RAG	0.26	1.79
KAV	0.24	1.77
GRG	0.73	1.74
SAF	0.21	-1.70
KKK	0.62	1.69
KRK	0.62	1.68
SKL	0.25	-1.68
IKL	0.20	1.67
EEE	0.68	1.67
KGG	0.40	1.65
RGG	0.75	1.63
ELE	0.43	-1.62
LKR	0.30	1.58
TGS	0.42	1.57
PYG	0.44	1.56
KNK	0.45	1.55
AIK	0.18	1.55
FQE	0.21	-1.54
RRI	0.21	1.54
VLF	0.04	-1.53
RSR	0.79	1.52
KKL	0.28	1.51
GAK	0.35	1.50
KLQ	0.28	-1.50
KKG	0.41	1.50
KVG	0.23	1.49
YGR	0.26	1.48
EIL	0.14	1.48
DIV	0.09	1.47
KEG	0.27	1.47
RTV	0.22	1.46
LAG	0.23	-1.44
DAK	0.33	1.44
EAE	0.55	-1.43
VKL	0.15	1.41
IGK	0.15	1.39
FIL	0.12	-1.39
SKK	0.54	1.39
FET	0.20	1.39
EAA	0.35	1.39
VYK	0.10	1.38
DFL	0.12	-1.38
KKR	0.57	1.37

Table S8: **Important tripetides from the *Salmonella* SVM model.** The top 50 important tripeptides from the *Salmonella* SVM model, i.e. the ones that contributed the most to the classification of *Salmonella* proteins into RBPs versus non-RBPs are ranked according to their absolute value of the SVM weight, computed as described in the Method section.

Tripeptide	frequency in disordered regions of RBPs	SVM weight
RRL	0.033	1.5
KVK	0.16	1.23
RAR	0.06	1.17
LRR	0	1.10
SRR	0.13	1.09
RKR	0.21	1.06
VTV	0.04	1.05
VKI	0	1.04
KRK	0.18	1.03
LTA	0.05	-1.03
LGQ	0	0.98
EVR	0.0	0.971
GRL	0.05	0.93
QLR	0	0.91
KKG	0.22	0.90
KTR	0.15	0.89
KAK	0.32	0.89
AGL	0	-0.88
RKT	0.19	0.88
KVE	0.06	0.88
ELE	0.11	0.88
RLL	0.012	0.88
KRT	0.17	0.87
RFV	0	0.86
GLA	0.032	-0.85
GKV	0.16	0.84
VEL	0.04	0.84
PVL	0.083	-0.83
SAL	0.04	0.83
PGA	0.11	0.82
VVE	0.21	0.82
KLQ	0	0.82
LLL	0.02	-0.82
ERG	0	0.80
LKG	0.04	0.80
DDA	0.07	-0.80
SGK	0.2	0.80
PFL	0	0.79
ASL	0	-0.79

IGA	0	-0.79
LKR	0.05	0.79
NKL	0.07	0.78
VFG	0	0.78
GRG	0.09	0.77
RGL	0.02	0.77
KIS	0	0.77
IKK	0.18	0.77
RER	0.16	0.76
RDG	0.07	-0.76
GVL	0	0.75

Table S9: **Important tripetides from the *E.Coli* SVM model.** The top 50 important tripeptides from the *E.Coli* SVM model, i.e. the ones that contributed the most to the classification of *E.Coli* proteins into RBPs versus non-RBPs are ranked according to their absolute value of the SVM weight, computed as described in the Method section.

Tripeptide	frequency in disordered regions of RBPs	SVM weight
KGK	0.07	1.58
IGA	0.00	-1.55
LRK	0.01	1.50
RKR	0.22	1.47
RTK	0.06	1.40
VTV	0.03	1.37
REA	0.12	1.33
PHP	0.09	1.32
LLG	0.03	1.27
RLL	0.01	1.25
LLK	0.02	1.25
KDV	0.11	1.21
RRL	0.07	1.20
KGP	0.25	1.20
VQV	0.15	1.19
KAK	0.30	1.18
SLT	0.00	-1.16
RKT	0.06	1.16
QRE	0.06	1.15
ADR	0.03	1.15
VNK	0.00	1.13
LRR	0.02	1.11
FGF	0.00	1.09
LEN	0.00	-1.08
GAI	0.07	-1.07
THL	0.03	1.07

VKA	0.22	1.05
HPK	0.00	1.05
KKK	0.24	1.04
VEL	0.03	1.04
CIV	0.00	1.03
GYR	0.10	1.03
AGF	0.05	1.02
PTR	0.28	1.02
IVD	0.00	1.01
VIK	0.09	1.01
VAD	0.00	1.00
SDL	0.00	-1.00
TAS	0.11	-1.00
VIG	0.00	-0.99
GRL	0.02	0.99
SAG	0.04	-0.99
KQK	0.04	0.99
LLL	0.00	-0.98
NAA	0.07	-0.98
APR	0.43	0.98
DAL	0.03	0.98
LML	0.00	-0.98
TRY	0.02	0.98
RLV	0.05	0.98

3 Supplementary Tables

We furthermore compiled tables with the prediction on the proteomes of all three species. These tables are in supplementary material and referenced as Table S13 - S15 for Human, *Salmonella* and *E.Coli*, respectively. These tables contain the Uniprot identifier of each protein, its name, the score from TriPepSVM, the inferred class of TriPepSVM (RBP or non-RBP), information on whether the protein was already annotated as RBP in Uniprot and the fraction of amino acids is oredered regions of the protein.
The proteins denoted as 'newly predicted' are not yet annotated as such in QuickGO, but predicted as RBPs by TriPepSVM.

We also compiled similar tables for the importance of all tri-peptides (Table S10 - S12) in which we report the the k-mers sorted by importance for the SVM decision boundary. The tables contain the k-mers, the number of times the k-mer was found in structurally ordered parts of proteins, the number of times it was found in disordered regions of proteins, its frequency in disordered regions and finally the feature weight of the k-mer.
